# Prevention of Surgical Site Infections: A Systematic Review of Cost Analyses in the Use of Prophylactic Antibiotics

**DOI:** 10.3389/fphar.2018.00776

**Published:** 2018-07-18

**Authors:** Abdul K. R. Purba, Didik Setiawan, Erik Bathoorn, Maarten J. Postma, Jan-Willem H. Dik, Alex W. Friedrich

**Affiliations:** ^1^Department of Health Sciences, University of Groningen, University Medical Center Groningen, Groningen, Netherlands; ^2^Department of Pharmacology and Therapy, Faculty of Medicine, Universitas Airlangga, Surabaya, Indonesia; ^3^Department of Medical Microbiology, University of Groningen, University Medical Center Groningen, Groningen, Netherlands; ^4^Unit of PharmacoEpidemiology & Pharmacoeconomics (PE2), Department of Pharmacy, University of Groningen, Groningen, Netherlands; ^5^Department of Pharmacology and Clinical Pharmacy, Faculty of Pharmacy, Universitas Muhammadiyah Purwokerto, Purwokerto, Indonesia; ^6^Department of Economics, Econometrics & Finance, Faculty of Economics & Business, University of Groningen, Groningen, Netherlands

**Keywords:** antibiotic prophylaxis, surgical wound infection, bacterial pathogens, health economic and outcome research, costs and cost analysis, systematic review

## Abstract

**Introduction:** The preoperative phase is an important period in which to prevent surgical site infections (SSIs). Prophylactic antibiotic use helps to reduce SSI rates, leading to reductions in hospitalization time and cost. In clinical practice, besides effectiveness and safety, the selection of prophylactic antibiotic agents should also consider the evidence with regard to costs and microbiological results. This review assessed the current research related to the use of antibiotics for SSI prophylaxis from an economic perspective and the underlying epidemiology of microbiological findings.

**Methods:** A literature search was carried out through PubMed and Embase databases from 1 January 2006 to 31 August 2017. The relevant studies which reported the use of prophylactic antibiotics, SSI rates, and costs were included for analysis. The causing pathogens for SSIs were categorized by sites of the surgery. The quality of reporting on each included study was assessed with the “Consensus on Health Economic Criteria” (CHEC).

**Results:** We identified 20 eligible full-text studies that met our inclusion criteria, which were subsequently assessed, studies had in a reporting quality scored on the CHEC list averaging 13.03 (8–18.5). Of the included studies, 14 were trial-based studies, and the others were model-based studies. The SSI rates ranged from 0 to 71.1% with costs amounting to US$480-22,130. Twenty-four bacteria were identified as causative agents of SSIs. Gram negatives were the dominant causes of SSIs especially in general surgery, neurosurgery, cardiothoracic surgery, and obstetric cesarean sections.

**Conclusions:** Varying results were reported in the studies reviewed. Yet, information from both trial-based and model-based costing studies could be considered in the clinical implementation of proper and efficient use of prophylactic antibiotics to prevent SSIs and antimicrobial resistance.

## Introduction

Surgical site infections (SSIs) reflect an important complication in modern healthcare (Berrios-Torres et al., 2017). As the surgical site is a potential port entry for exogenous organisms, it poses an immediate threat to the body and infections lead to prolonged wound healing (Mangram et al., 1999; Berrios-Torres et al., 2017). The preoperative phase is considered the most crucial period of a surgical procedure in which the goal is to reduce the bacterial load surrounding the incision area. Using antibiotics prior to surgical incision is considered to be effective in preventing SSIs, which are among the most common preventable post-surgery complications involving healthcare-associated infections (HAIs) (Mangram et al., 1999; Umscheid et al., 2011). A parenteral prophylaxis agent spectrum with corresponding potential bacteria on particular sites of surgery has been recommended recently to reduce SSI rates efficiently (Berrios-Torres et al., 2017). In contrast, some preoperative procedures, such as hair removal and mechanical bowel preparation are considered today to be inefficient at reducing SSIs (Leaper et al., 2008; Anderson et al., 2014).

In the US, SSIs were identified in ~1.9% of 849,659 surgical procedures in 43 states from 2006 to 2008 (Mu et al., 2011). The economic burden of SSIs should be taken into account in the use of prophylactic antibiotics. In the US in 2010, more than 16 million surgical procedures were performed (CDC, 2010). The annual costs of SSIs amounted to approximately US$3 billion in 2012, having increased from an estimated US$1.6 billion cost attributable to SSIs in 2005 (de Lissovoy et al., 2009; Zimlichman et al., 2013). In low-and middle-income countries, SSI rates doubled from 5.6 to 11.8 in 100 surgical patients between 1995 and 2008 (Allegranzi et al., 2011). The reporting of cost and effectiveness in infectious disease presents a crucial topic, ideally supported by updated antimicrobial resistance data. Notably, economic analyses can be differentiated into trial-based—directly linked to a trial that often also already comprises part of the economic variables—and model-based studies with information gathered from various sources and integrated into a health-economic model. The aim of this study is to present recent evidence from trial-based and model-based costing studies and analyze the methodologies used in economic evaluations of prophylactic antibiotics in SSI prevention. In addition, the study comprehensively analyzes the quality of the included studies and local epidemiology of pathogen-causing SSIs.

## Materials and methods

This review was registered in PROSPERO with number CRD42017076589. This study was designed according to the Preferred Reporting Items for Systematic Reviews and Meta-Analyses (PRISMA) statement (Moher et al., 2009).

### Search strategy

We searched the updated relevant evidence from PubMed and EMBASE databases. To consider changes over time in inflation rates, value of money, and patterns in microbial causes of SSIs and contemporary antimicrobial susceptibility, we initially searched a 10-year period (2006-2016) which we later updated to 31 August 2017. The search used search terms or phrases represented in Medical Subject Headings (MeSH) with the operator “tiab” for PubMed. Subsequently, the terms or phrases used in PubMed were translated to the EMBASE database by using strings and the symbols “ab,ti.” To refine the result, we employed a search strategy using the Boolean operator “OR” within sequences of terms with close or similar meanings and “AND” for one or more sequences of terms which contained completely different meanings. Whole terms and phrases for either PubMed or Embase were identified by two persons (AKRP and KS) who dealt with the search strategy.

### Study selection

Trial-based and model-based studies that examined the clinical benefits and costs related to the uses of prophylactic antibiotics for SSIs were considered as eligible for inclusion in this review. Therefore, we developed criteria to identify the eligible studies which contained economic analysis and followed the defined PICO-approach (Patient or Problem, Intervention, Control, and Outcomes). Concerning the patient (P), patients undergoing all types of surgical procedures were included. There was no restriction on age or gender. For both the intervention (I) and comparison (C), this review included studies concerning the utilization of antibiotic prophylaxis administered intravenously, orally, or locally to prevent SSI. Other terms of post-surgical infections such as wound infections and sternal wound infections (SWIs) were included. We excluded studies mainly evaluating comparisons of the use of antiseptic, pharmaceutical care interventions or guideline adherence issues. For the outcomes (O), we included studies evaluating both SSI rates and cost. Eventually, the integrated results of searches were restricted to English full-text studies. Studies issued as commentary, editorials, research protocols or reviews were excluded.

### Data extraction

Two authors (AKRP and DS) independently assessed all included papers. Any disagreements between those authors were discussed with a third author (JWD) until the discrepancies were resolved by consensus. Fields of the extracted data included the authorship, year of publication, journal, country, type of surgery, wound categorization, gender, age, sample size, outcomes, prophylactic antibiotics, SSI rates, timing for the prophylactic strategy, follow-up, and length of stay. To address the outcome from a microbiology perspective, we extracted the pathogens based on the sites of the surgery, antimicrobial susceptibility, and their resistance rates. Furthermore, we grouped the types of SSIs based on the definitions and classifications of SSIs from the Centers for Disease Control and Prevention (CDC) (Horan et al., 1992).

### Cost analysis and data synthesis

We categorized the methodology on the health-economic analyses and outcomes for each eligible study according to four approaches (Drummond et al., 2015). The first was cost minimization analysis (CMA), which represents a straightforward method to identify the costs of different alternatives with estimated equal health outcomes of the interventions. The second was cost effectiveness analysis (CEA) where the outcomes are expressed in a natural unit of health including the number of patients with clinical improvement of an infectious disease or life-years gained. The third was a cost benefit analysis (CBA), in which the interventions are made comparable in terms of benefit and cost with all aspects being expressed in financial units. The last was cost utility analysis (CUA), which includes utility estimates potentially representing preferences for health outcomes, reporting quality-adjusted life years (QALYs) or alternatively disability-adjusted life years (DALYs). Furthermore, for the cost types, we took into account cost perspectives with components of (1) direct costs such as costs for prophylactic antibiotics, hospitalization, side-effects, and antimicrobial resistance, and (2) indirect costs including costs of loss of productivity. We made costs comparable among individual studies using currency conversions to US$ and corrections for inflation rates. We calculated inflation rates based on the 2015 annual GDP growth index in the World DataBank for each respective country (The World Bank, 2015). If the individual article did not state the actual year for the cost analyses, we made the assumption that the year of the cost estimate was the same as the last year of data collection.

### Quality assessments

We used the Consensus on Health Economic Criteria (CHEC) list to assess the quality of reporting of the health economic outcomes, including potential bias in individual studies (Evers et al., 2015). This CHEC instrument comprises a 19-item list that relates to study design (4 items), time horizon, actual perspective, cost evaluation (5 items), outcome measurements (3 items), discounting, conclusion, generalization, conflict of interest, and ethical issues (Husereau et al., 2013). These items can be conceived to reflect minimum requirements for health economic papers. We scored one point for “yes,” indicating an item to be satisfied. Marks of “unclear” and “no” were scored half a point and zero respectively. Therefore, the minimum and maximum scores for the individual studies were in a range of 0 to 19.

## Results

### Search results

**Figure 1:**
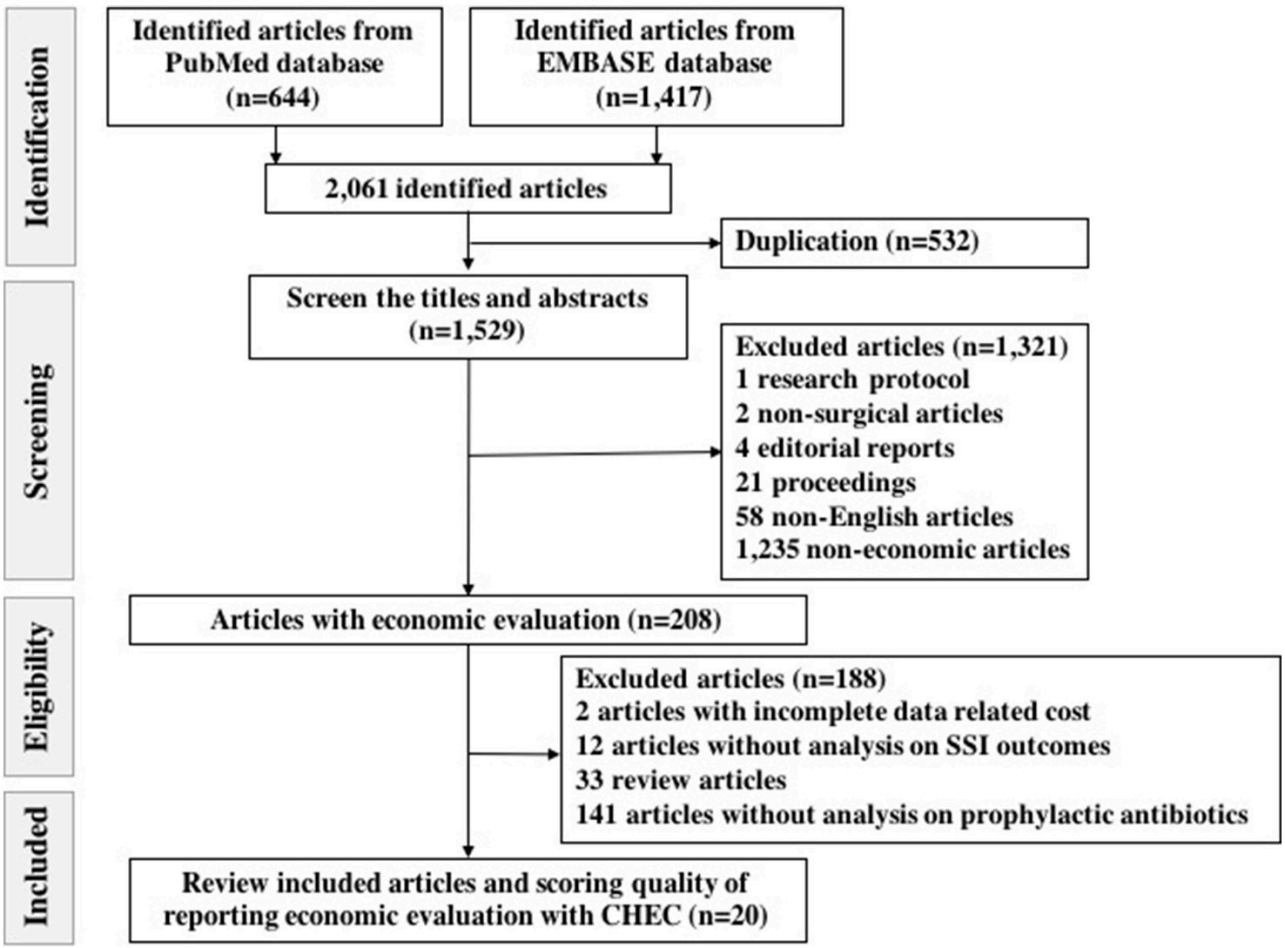
Flow chart of search strategy on identifying eligible and included studies with standard report of the Preferred Reporting Items for Systematic Reviews and Meta-Analyses (PRISMA).

This review initially identified a total of 644 and 1,417 articles from PubMed and Embase respectively. A comprehensive listing of the searches in both PubMed and Embase can be found in Tables S1, S2. After removing duplications, we screened 1,529 titles and abstracts. Subsequently, we excluded 1,321 articles for the reasons listed in the Materials and Methods section. Eventually, we assessed 208 eligible full-text studies of which we excluded 188 because of being reviews, having incomplete data related to costs and lack of presenting on the outcomes of prophylactic antibiotic uses and SSI incidence (Table S3). A total of 20 articles remained according to the inclusion criteria and were extracted systematically for further analyses (Chaudhuri et al., 2006; Dhadwal et al., 2007; Alekwe et al., 2008; Wilson et al., 2008; Kosus et al., 2010; Patil et al., 2011; Courville et al., 2012; Gulluoglu et al., 2013; Merollini et al., 2013; Emohare et al., 2014; Matsui et al., 2014; Theologis et al., 2014; Joshi et al., 2016). A flow chart of the search is shown in Figure 1.

### General characteristics of included studies

The general characteristics of the included studies are presented in Table 1. For the further characteristics of the 20 included studies, the most studies came from North-America (Wilson et al., 2008; Courville et al., 2012; Emohare et al., 2014; Matsui et al., 2014; Singh et al., 2014; Theologis et al., 2014; Graves et al., 2016; Lewis et al., 2016), followed by Asia (Kosus et al., 2010; Patil et al., 2011; Gulluoglu et al., 2013; Ozdemir et al., 2016), Europe (Chaudhuri et al., 2006; Dhadwal et al., 2007; Elliott et al., 2010; Joshi et al., 2016), Africa (Alekwe et al., 2008; El-Mahallawy et al., 2013), Australia (Merollini et al., 2013), and South America (Ceballos et al., 2017). The reviewed articles concerned 14 trial-based studies (Chaudhuri et al., 2006; Dhadwal et al., 2007; Alekwe et al., 2008; Wilson et al., 2008; Kosus et al., 2010; Patil et al., 2011; El-Mahallawy et al., 2013; Gulluoglu et al., 2013; Emohare et al., 2014; Matsui et al., 2014; Theologis et al., 2014; Joshi et al., 2016; Lewis et al., 2016; Ozdemir et al., 2016) and 6 model-based studies (Elliott et al., 2010; Courville et al., 2012; Merollini et al., 2013; Singh et al., 2014; Graves et al., 2016; Ceballos et al., 2017). Table 2 presents the baseline characteristics of the included studies. Moreover, six trial-based studies were performed as a formal randomized controlled trial (RCT) with the number of patients involved in the studies ranging between 50 and 1,196 (Chaudhuri et al., 2006; Alekwe et al., 2008; Kosus et al., 2010; El-Mahallawy et al., 2013; Gulluoglu et al., 2013; Matsui et al., 2014).

**Table 1:** General characteristics of the 20 included articles.

**Characteristics**	**Included articles *n* (%)**
**REGION**	
Africa	2 (10)
Asia	4 (20)
Australia	1 (5)
Europe	4 (20)
North America	8 (40)
South America	1 (5)
**TYPE OF SURGERY**	
Cardiothoracic	2 (10)
General	5 (25)
Neurosurgery	2 (10)
Obstetric gynecology	2 (10)
Oncology	3 (15)
Orthopedic	6 (30)
**TYPE OF ECONOMIC EVALUATION**	
CBA	0
CEA	3 (15)
CMA	15 (75)
CUA	2 (10)

*CMA, cost minimization analysis; CBA, cost benefit analysis; CEA, cost effectiveness analysis; CUA, cost utility analysis*.

**Table 2:** Baseline characteristics of included studies regarding country, type of surgery, prophylactic antibiotics, gender, mean age, number of subjects, outcome measure, study design, antibiotic susceptibility, and prophylactic timing.

**Author, year**	**Country**	**Type of surgery**	**Prophylactic antibiotic**		**Gender**	**Mean age (years)**	**Number of subject**	**Outcome measure**	**Study design**	**Antibiotic susceptibility**	**Prophylactic timing**

			**Study group**	**Control group**							
**GENERAL SURGERY**											
Chaudhuri et al., 2006	Germany	Excision of pilonidal sinuses	Metronidazole 500 mg i.v.	Cefuroxime 1.5 g i.v.+ metronidazole 500 mg i.v.	F and M	27	SG: 25; CG: 25	Infection related wound complications, total costs	Double-blinded RCT	NA	30 min prior to incision and continued to day 5 for multi-drug
Wilson et al., 2008	United States	Elective colorectal surgery	Ertapenem 1 g i.v.	Cefotetan 2 g i.v.	F and M	SG: 61.3; CG: 60.2	SG: 338; CG: 334	SSIs, antibiotic use, anastomotic leak of the bowel, cost per dose, direct medical costs	Observational study: analysis from PREVENT trial (Itani et al., 2006)	NA	30 min prior to incision
Matsui et al., 2014	United States	Laparoscopic cholecystectomy	Cefazolin sodium 1 g i.v.	Without prophylactic antibiotics	F and M	Over 65: 197 patients (SG) and 202 patients (CG)	SG: 518; CG: 519	Postoperative infections (SSIs, distant infections), hospital stay, antibiotic costs, direct medical costs	RCT	NA	Before skin incision
Singh et al., 2014	United States	Abdominal surgery	Triclosan-coated sutures	Without triclosan-coated sutures	NA	All ages included	1,000 individuals	Cost saving of triclosan-coated sutures when SSI-risks were 5, 10, and 15%	Model-based study	NA	NA
Ozdemir et al., 2016	Turkey	Elective colorectal resections	Cefazolin 1 g i.v. and metronidazole 500 mg i.v. plus metronidazole 4 g p.o. + gentamicin 480 mg p.o.	Cefazolin 1 g i.v. and metronidazole 500 mg i.v.	F and M	SG: 58; CG: 59	SG: 45; CG:45	SSIs, length of hospital stays, cost saving	Retrospective study	NA	Start from during anesthesia induction to 5 days post-operation
**ORTHOPEDIC**											
Elliott et al., 2010	United Kingdom	Primary hip arthroplasty	Cephalosporin	Vancomycin or combination of vancomycin and cephalosporin	M	65	1,770	SSIs, MRSA infection, length of stay, mortality, utility in QALYs, costs	Model-based study	NA	NA
Courville et al., 2012	United States	Total hip and knee arthroplasty	Nasal mupirocin	Without mupirocin	NA	Hypothetical cohort of 65-year-old	NA	SSIs, utility in QALYs, costs	Model-based study	NA	NA
Merollini et al., 2013	Australia	Total hip arthroplasty	Antibiotic prophylaxis	No antibiotic prophylaxis, antibiotic-impregnated cement, laminar air operating rooms.	NA	65 years	30,000 hypothetical cohorts	SSIs, utility in QALYs, length of stay, mortality	Decision model and cost effectiveness analysis	NA	NA
Theologis et al., 2014	United States	Thoracolumbar adult deformity reconstruction	Intravenous antibiotics and vancomycin powder 2 g	Intravenous antibiotics	F and M	SG: 62.4; CG: 60.0	SG: 151; CG: 64	SSIs, utility in QALYs, add-on impregnated prophylactic antibiotic cost, cost saving	Retrospective cohort study	NA	NA
Graves et al., 2016	United States	Primary hip replacement	No systemic antibiotics	Systemic antibiotics	NA	NA	77,321	Deep infections, utility in QALYs, costs	Model-based study	NA	NA
Ceballos et al., 2017	Colombia	Lower limb amputation	Cefazolin, cephalothin, cefotaxime, cefoxitin, cefuroxime	Non-prophylactic antibiotics	NA	NA	10,000 simulations	Decision analytic model of superficial and deep infections, healing, sepsis, re-amputation, mortality, costs	Model-based study	NA	NA
**NEUROSURGERY**											
Emohare et al., 2014	United States	Posterior spinal surgery	Cefazolin i.v. and vancomycin powder 1 g intra-wound	Cefazolin i.v.	F and M	SG: 53.7; CG: 58.2	SG: 96; CG: 207	SSIs, intra-wound costs, direct costs	Retrospective cohort study	NA	NA
Lewis et al., 2016	United States	Cranial surgery and subdural or subgaleal drains	PPSAs of Cefazolin and Vancomycin	Non-PPSAs	F and M	SG: 59; CG: 57	SG: 105; CG: 80	SSIs, direct costs and cost saving	Retrospective study	NA	NA
**CARDIOTHORACIC SURGERY**											
Dhadwal et al., 2007	United Kingdom	Coronary artery bypass grafting surgery	Rifampicin 600 mg p.o., gentamicin 2 mg/kg i.v. and vancomycin 15 mg/kg i.v.	Cefuroxime 1.5 g i.v.	F and M	SG: 62.8; CG: 65.4	SG: 87; CG: 99	SWIs, antibiotic and hospital costs	Double-blinded RCT	NA	Rifampicin 1 h preoperatively, gentamicin and vancomycin after anesthesia induction
Joshi et al., 2016	United Kingdom	Cardiac surgery in high risk of SWI	Gentamicin-impregnated collagen sponges	Without gentamicin-impregnated collagen sponges	NA	NA	1251	SWI incidence, median postoperative cost, annual additional cost for SWIs and the gentamicin-impregnated collagen sponges	Observational study	NA	NA
**OBSTETRIC GYNECOLOGICAL SURGERY**											
Alekwe et al., 2008	Nigeria	Elective cesarean section	Ceftriaxone 1 g i.v.	Ampicillin/ cloxacillin 1 g q.i.d. i.v., gentamicin 80 mg t.i.d. i.v. and metronidazole 500mg t.i.d. i.v.	F	SG: 33.53; CG: 32.08	SG: 100; CG: 100	Infectious morbidity, endometritis, UTI, febrile morbidities, wound infections, duration of hospital stay, antibiotic costs	RCT	NA	NA
Kosus et al., 2010	Turkey	Cesarean section	Ceftriaxone 1 g i.v. and rifamycin 250mg	Ceftriaxone 1 g i.v.	F	SG: 28.4; CG: 26.8	SG: 596; CG: 600	SSI rates, cost for rifamycin and SSI treatments	RCT	NA	NA
**ONCOLOGIC SURGERY**											
Patil et al., 2011	India	Head and neck onco-surgeries	Single antibiotic of cefazolin, or ciprofloxacin, or cefprozil, or clindamycin	Combination antibiotics of cefazolin and metronidazole, or clindamycin and gentamicin, or ampicillin/ cloxacillin, or moxifloxacin and metronidazole, or ciprofloxacin and metronidazole, or cefprozil and metronidazole	F and M	NA	50	Post-operative wound infections, costs for prophylactic antibiotics and post-operative antibiotics	Observational study	NA	NA
Gulluoglu et al., 2013	Turkey	Breast cancer surgery	Ampicillin-sulbactam 1 g i.v.	Without prophylactic antibiotics	F	SG: 58.8; CG: 58.2	SG: 187; CG: 182	SSIs, time to SSIs, culture results, adverse reactions due to antibiotics, mean SSI-related costs	RCT	NA	NA
El-Mahallawy et al., 2013	Egypt	Cancer surgery (bladder, stomach, colon, rectum)	Penicillin G sodium 4,000,000 IU i.v. and gentamicin 80 mg i.v.	Clindamycin 600 mg i.v. and amikacin 500 mg i.v.	F and M	* ≤* 40 years: 72; >40 years: 128	SG: 100; CG: 100	SSI incidence and cost for prophylactic antibiotics	RCT	NA	NA

*CG, control Group; F, female; i.v., intravenous; M, male; MRSA, Methicillin-resistance Staphylococcus aureus; NA, not available; p.o., per oral; PPSAs, prolonged prophylactic systemic antibiotics; QALYs, quality adjusted life years; q.i.d., quarter in die (four times a day); RCT, randomized control trial; SG, study group; SSIs, surgical site infections; SWIs, sternal wound infections; t.i.d., ter in die (three times a day); UTI, urinary tract infection*.

### Antibiotic prophylaxis in general surgery

Five included studies analyzed the cost and effectiveness of antibiotic prophylaxis in general surgery (Chaudhuri et al., 2006; Wilson et al., 2008; Matsui et al., 2014; Singh et al., 2014; Ozdemir et al., 2016). The types of surgery were pilonidal sinus excision (Chaudhuri et al., 2006), elective colorectal surgery (Wilson et al., 2008; Ozdemir et al., 2016), laparoscopic cholecystectomy (Matsui et al., 2014), and general abdominal surgery (Singh et al., 2014). The included studies indicated that new generation antibiotics generated economic benefit in SSI prevention. An observational study reported that the use of ertapenem in elective colorectal surgery achieved cost savings of roughly US$2,200 per patient compared with cefotetan (Wilson et al., 2008). The secondary costs due to selection regarding resistance were not taken into account in this study and would need to be assessed in future studies. Another study showed that triclosan-coated sutures seemed to be cost saving and effective at reducing SSI rates from the hospital, payer, and societal perspectives (Singh et al., 2014). However, no long-term data on tissue-toxicity and possible triclosan-induced inflammatory response was included in this study. In addition, single prophylactic antibiotics and both oral or intravenous administration were demonstrated to have a positive impact on reducing SSI rates and medical costs in general surgery (Chaudhuri et al., 2006; Matsui et al., 2014).

### Antibiotic prophylaxis in orthopedic surgery

Various studies modeled economic and clinical impacts from the societal and healthcare perspectives of patients undergoing total hip arthroplasty (THA), total knee arthroplasty (TKA), and lower limb amputation (Elliott et al., 2010; Courville et al., 2012; Merollini et al., 2013; Graves et al., 2016; Ceballos et al., 2017). Economic analysis on the implementation of the use of nasal mupirocin to prevent deep SSI of *Staphylococcus aureus* in THA and TKA showed that mupirocin was more cost-effective compared to non-preoperatively administered mupirocin with incremental cost-effectiveness ratios (ICERs) at US$380.09/QALY and US$517.16/QALY for THA and TKA respectively (Courville et al., 2012). Vancomycin has also been taken into account as an intra-wound antibiotic, with SSI rates of 3 and 11% were identified in the group with and without 2 g vancomycin powder respectively. Clinically and economically, these percentages were considered to reflect a significant impact with cost savings of US$2,762 per operative procedure at day 90 post-surgery (Theologis et al., 2014). Furthermore, two studies addressed that prophylactic intervention was dominant over no prophylactic antibiotics on SSI rates and cost reductions in total hip arthroplasty and lower limb amputation (Merollini et al., 2013; Ceballos et al., 2017).

### Antibiotic prophylaxis in neurosurgery

Two cost-minimization studies on neurosurgery concerned intra-wound vancomycin and Prolonged Prophylactic Systemic Antimicrobials (PPSAs) (Emohare et al., 2014; Lewis et al., 2016). Firstly, a cohort study, for the purpose of reimbursement to the hospital for SSI costs, evaluated the cost savings achieved by adding intra-wound vancomycin powder as prophylactic therapy to standard intravenous cefazolin in patients who underwent spinal surgery. No SSIs were reported in patients who received intra-wound vancomycin, whereas seven out of 207 patients who were given only cefazolin developed SSIs at a cost of US$2,879 per patient (Emohare et al., 2014). Secondly, a retrospective study looked into the duration of prophylactic antibiotic use in cranial surgery and subdural or subgaleal drains. Continuous prophylactic antibiotics or PPSAs were considered costly compared with non-PPSA treatment in the operation, which saved US$93,195 per patient (Lewis et al., 2016).

### Antibiotic prophylaxis in cardiothoracic surgery

Two included RCTs and an observational study evaluated the clinical and economic impact of antibiotics for prevention of SWIs in coronary surgery. First, one RCT study reported that the use of triple antibiotics of rifampicin gentamicin, and vancomycin for SSI prophylaxis could reduce the total cost of treatment by US$4,521 per patient compared to single prophylaxis of cefuroxime (Dhadwal et al., 2007). Second, an observational study on Gentamicin-impregnated Collagen Sponges (GCS) to prevent SSIs in cardiac surgery noted a unit cost of GCS of roughly US$129 per patient. Nevertheless, in their cost analysis, they remarked that GCS provided no economic benefit in reducing SSI incidence by 50% SSI in a 2-year period (Joshi et al., 2016).

### Antibiotic prophylaxis in obstetric and gynecological surgery

In obstetric and gynecological surgery, two included RCT-based studies analyzed SSI incidence and performed an economic impact analysis of ceftriaxone prophylactic in delivery through cesarean section. The first RCT compared single-dose prophylactic ceftriaxone to a triple drug combination of ampicillin/cloxacillin, gentamicin, and metronidazole. Notwithstanding the economic benefit of a single dose of ceftriaxone compared with the combination regimen, the SSI rates were between 7% with ceftriaxone and 8% with the triple drug treatments (Alekwe et al., 2008). The second RCT was carried out on the implementation of subcutaneous rifamycin as add-on therapy for prophylactic ceftriaxone. Twelve allocated subjects for the standard prophylactic were followed up with SSI, and in these cases, the total cost related to SSI treatment amounted to US$483 per patient. On the other hand, no patient developed SSI by the end of the follow-up period in the intervention group (Kosus et al., 2010).

### Antibiotic prophylaxis in oncology surgery

Three included studies concerned different operative procedures in oncology surgery for malignancies of the breast, head-neck, bladder, stomach, colon, and rectum (Patil et al., 2011; El-Mahallawy et al., 2013; Gulluoglu et al., 2013). An observational study in surgery for head and neck cancer by Patil et al. (2011) conveyed that no significant difference was indicated in the total cost between single and combination antibiotics. On the other hand, an RCT study on breast cancer surgery by Gulluoglu et al. (2013) presented that antibiotic prophylaxis with intravenous ampicillin-sulbactam was more cost saving and effective compared to no prophylaxis, resulting in a 9% reduction in the SSI rate and a cost reduction of US$11 per patient. Moreover, another RCT on abdominal cancer by El-Mahallawy et al. (2013) indicated cost savings with the combination of penicillin and gentamicin over using clindamycin and amikacin. Table 3 compares the included studies on reporting cost analysis. In addition, the SSI rates and the cost ranges of each surgical procedure are presented in Table 4.

**Table 3:** Comparisons of included studies on reporting of cost index year, cost analysis method, cost perspective, and costs (adjusted to US$ at 2015 prices).

**Study, year of publication**	**Cost index year**	**Cost analysis method**	**Cost perspective**	**Adjusted costs in US$**
**GENERAL SURGERY**				
Chaudhuri et al., 2006	2006	CMA	NA	Total cost in group with a single-dose Metronidazole: US$11.53 per patient Total cost for SSI complications: US$813.25 per patient
Wilson et al., 2008	2005	CMA	NA	Cost per dose of ertapenem: US$47.86 per patient Cost per dose of cefotetan: US$29.78 per patient Direct medical cost in group with etapenem prophylaxis: US$16,433.89 per patient Direct medical cost in group with cefotetan prophylaxis: US$18,812.66 per patient
Matsui et al., 2014	2013	CMA	NA	Cost for antibiotics in group with cefazolin: US$25.73 per patient; Cost for antibiotics in group without prophylactic: US$8.37 per patient; Direct medical cost in group with cefazolin: US$791.59 per patient; Direct medical cost in group without prophylactic: US$859.58 per patient.
Singh et al., 2014	2013	CMA	Healthcare, payer and societal perspective	For 15% SSI risk, triclosan-coated suture saved: - US$4,232.27 – 14,394.25 per patient (Hospital perspective) - US$4,256.99 – 14,725.91 per patient (Payer perspective) - US$41,330.81 – 54,841.32 per patient (Societal perspective)
Ozdemir et al., 2016	2016	CMA	NA	Total hospital cost: - In group with cefazolin and metronidazole intravenously plus metronidazole and gentamicin orally: US$2,699 per patient - In group with cefazolin and metronidazole intravenously: US$4,411 per patient
**ORTHOPEDIC**				
Elliott et al., 2010	2005	CUA	Societal perspective	Total cost per QALY for SSI-treatments - In group with vancomycin prophylactic: US$1,417.78/QALY - In group with cephalosporin prophylactic: US$1,418.01/QALY - In group with combination prophylactic: US$1,421.48/QALY
Courville et al., 2012	2005	CEA	Societal perspective	Average cost per QALY:
				Total hip arthroplasty: - Treated with mupirocin: US$34,990.65/QALY - Treated with mupirocin and screened positive for *S.aureus*: US$35,308.54/QALY - Without mupirocin: US$35,370.74/QALY
				Total knee arthroplasty: - Treated with mupirocin: US$41,368.18/QALY - Treated with mupirocin and screened positive for *S.aureus*: US$41,775.92/QALY - Without mupirocin: US$41,885.34/QALY
Merollini et al., 2013	2011	CEA	Healthcare perspective	ICER non-prophylactic compared with prophylactic antibiotics: US$9,917.14/QALY-lost Add-on antibiotic-impregnated prophylaxis saving US$4,164.81/QALY-gained
Theologis et al., 2014	2009	CMA	NA	Cost for vancomycin powder: US$38.30 per operative procedure Total cost in group with vancomycin powder: US$78,745.18 per operation Total cost in group without vancomycin powder: US$71,514.31 per operation Cost saving using vancomycin powder: US$276,174.26 per 100 operative procedures
Graves et al., 2016	2012	CUA	Healthcare perspective	With the reference of non-systemic antibiotics + plain cement + conventional ventilation (T0), ICERs of T1 to T8: - T1: US$120,989.52/QALY - T2: US$83,904.20/QALY - T3: US$75,533.82/QALY - T4: US$88,054.96/QALY - T5: US$95,765.38/QALY - T6: US$44,615.47/QALY - T7: US$63,185.13/QALY - T8: US$21,302/QALY
Ceballos et al., 2017	2014	CEA	Healthcare perspective	Incremental cost between non-prophylactic and prophylactic group: US$1,245.83 per patient
**NEUROSURGERY**				
Emohare et al., 2014	2012	CMA	NA	Cost for intra-wound vancomycin: US$12.46 per patient Direct medical cost in group without intra-wound vancomycin: US$2,879.02 per patient
Lewis et al., 2016	2015	CMA	NA	Direct cost for PPSAs: US$887.50 per patient Cost saving for Non-PPSAs: US$93,194.63 per patient
**CARDIOTHORACIC SURGERY**				
Dhadwal et al., 2007	2004	CMA	NA	Cost for prophylactic antibiotics: - In the group with single prophylactic antibiotic: US$540.18 per patient - In the group with combination prophylactic antibiotics: US$425.95 per patient
				Total hospital costs: - In the group with single prophylactic antibiotic: US$22,130.53 per patient - In the group with combination prophylactic antibiotics: US$17,609.24 per patient
Joshi et al., 2016	2013	CMA	NA	Median cost without SSI: US$15,502.72 per patient
				Median additional cost for SSI treatments: US$7,835.59 per patient
				Cost for the GCS: US$128.96 per patient
				Total annual additional costs in reducing SSI incidence by 50% - Without GCS: US$70,523.90 per patient - With GCS: US$115,924.59 per patient
**OBSTETRIC GYNECOLOGICAL SURGERY**				
Elliott et al., 2010	2008	CMA	NA	Costs for antibiotics: - In group with single prophylactic antibiotic: US$10.61 per patient - In group with combination prophylactic antibiotics: US$16.52 per patient
Courville et al., 2012	2007	CMA	NA	The price of rifamycin: US$1.58 per patient
				Mean cost for SSI treatments: US$482.59 per patient
**ONCOLOGIC SURGERY**				
Patil et al., 2011	2007	CMA	NA	Costs in group with single antibiotic: - Prophylactic antibiotic costs: US$7.22 per patient - Post-surgical antibiotic costs: US$79.76 per patient - Total antibiotics costs: US$86 per patient
				Costs in group with combination of prophylactic antibiotics: - Prophylactic antibiotic costs: US$12.13 per patient - Post-surgical antibiotics costs: US$82.79 per patient - Total antibiotics costs: US$94.92 per patient
Gulluoglu et al., 2013	2010	CMA	NA	Costs for SSI treatments: - In group with prophylactic antibiotics: US$9.18 per patient - In group without prophylactic antibiotics: US$21.93 per patient
El-Mahallawy et al., 2013	2013	CMA	NA	Direct cost in group with penicillin G sodium and gentamicin: US$3.26 per patient Direct cost in group with clindamycin and amikacin: US$17.39 per patient

*CMA, cost minimization analysis; GCS, Gentamicin collagen sponges; ICER, incremental cost-effectiveness ratio; NA, not available; PPSAs, prolonged prophylaxis systemic antimicrobials; T1, systemic antibiotics + plain cement + conventional ventilation; T2, non-systemic antibiotics + plain cement + laminar airflow; T3, systemic antibiotics + plain cement + laminar airflow; T4, non-systemic antibiotics + antibiotic-impregnated cement + conventional ventilation; T5, systemic antibiotics + antibiotic-impregnated cement + conventional ventilation; T6, systemic antibiotic + antibiotic-impregnated cement + laminar airflow; T7, systemic antibiotics + antibiotic-impregnated cement + ventilation + body exhaust suit; T8, systemic antibiotics + antibiotic-impregnated cement + laminar ventilation + body exhaust suit; THA, total hip arthroplasty; TKA, total knee arthroplasty; US$, the United States Dollars*.

**Table 4:** Comparison of selected studies on reporting of SSI classification, SSI rate, statistical significance, timing at the identification of the SSI and length of hospitalization.

**Study, year of publication**	**SSI classification**	SSI rate^*^	**Statistical significance**	**Timing SSI identified**	**Length of hospitalization (days)**
**GENERAL SURGERY**					
Chaudhuri et al., 2006	Superficial	Metronidazole: 11(44%) Cefuroxime and metronidazole: 3(12%)	*p-*value: 0.9, < 0.0001 and < 0.03 at week 1, 2, and 4 respectively	Week 1,2, and 4	NA
Wilson et al., 2008	Superficial, deep, organ space	Ertapenem: 62(18.3%) Cefotetan: 104(31.1%)	CI95% absolute difference: −19.5 to 6.5	Week 4	Ertapenem: 9; Cefotetan: 11.6
Matsui et al., 2014	NA	Cefazolin: 4(0.8%) Without prophylactic antibiotic: 19(3.7%)	*p*-value: 0.001	Day 1 and or day 2 postoperative	Cefazolin: 3.69 No antibiotic: 4.07
Singh et al., 2014	Superficial and deep SSI	An assumption of SSI-risk for the triclosan coated sutures treatment: 5-20%	NA	30–90 days	NA
Ozdemir et al., 2016	Superficial, deep and organ space	Combination of intravenous prophylaxis (cefazolin and metronidazole) and oral prophylaxis (metronidazole and gentamicin): 16(35.6%) Intravenous prophylaxis only (metronidazole and gentamicin): 32(71.1%)	*p*-value < 0.001	30 days	Intravenous only: 14.2 Combination of intravenous and oral prophylaxis: 8.1
**ORTHOPEDIC**					
Elliott et al., 2010	Superficial and deep/joint	Vancomycin group: 2(0.4%) infected by MRSA and 41(9.1%) infected by others Cephalosporin group: 7(1.6%) infected by MRSA and 32(7.4%) infected by others	NA	30 days	NA
Courville et al., 2012	Deep	Probability among Mupirocin-treated carriers: 1.3% Probability among non-Mupirocin and non-carriers: 0.58%	NA	Time horizon: 1 year	NA
Merollini et al., 2013	Deep	Incremental SSI incidence: - In non-prophylactic antibiotic group over prophylactic group: 230 cases - In add-on antibiotic-impregnated cement over antibiotic prophylaxis: prevented 46 cases	NA	Time horizon: 30 years	NA
Theologis et al., 2014	NA	Intravenous antibiotics and vancomycin powder: 4(2.6%) Intravenous antibiotic only: 7(10.9%)	*P*-value: 0.01	90 days	NA
Graves et al., 2016	Deep	T0: 1887 cases T1: 870 cases T2: 670 cases T3: 721 cases T4: 950 cases T5: 406 cases T6: 666 cases T7: 905 cases T8: 1126 cases	CI95%: T0: 1253–2621 T1: 345–1655 T2: 90–1937 T3: 192–1589 T4: 286–2059 T5: 90–964 T6: 101–2017 T7: 77–2499 T8: 143–2827	Time horizon: 30 days for non-implant and 1 year for implant procedures	NA
Ceballos et al., 2017	Superficial and deep	Prophylactic antibiotic: 62(16.2%) Non-prophylactic antibiotic: 44(38.3%)	NA	NA	NA
**NEUROSURGERY**					
Emohare et al., 2014	Superficial [study group: 5 (5%); control group: 5(2%)]; Deep [study group: 0; control group:7(3%)]	Cefazolin and vancomycin: 0 out of 96 Cefazolin: 7(3.4%)	NA	20–22 months	NA
Lewis et al., 2016	Superficial and deep	PPSAs: 2(1.9%) Non-PPSAs: 1(1.3%)	Deep SSI: *p* = 1.00 Superficial SSI: *p* = 0.77	90 days	PPSAs and non-PPSAs: 5
**CARDIOTHORACIC SURGERY**					
Dhadwal et al., 2007	Superficial, deep, organ space	Rifampicin + gentamicin + vancomycin: 8(9.2%) Cefuroxime: 25(25.3%)	NA	Day 90	Triple antibiotics: 9.1 Single antibiotic: 12
Joshi et al., 2016	Deep and superficial sternal wound infection	18(1.4%) diagnosed as SWI in a two-year period	NA	NA	Wards: 5 (non-SWI) and 12.7 (SWI) ICU: 2.5 (non-SWI) and 3 (SWI)
**OBSTETRIC GYNECOLOGICAL SURGERY**					
Alekwe et al., 2008	Deep	Ceftriaxone: 7(7%) Amplicox + gentamicin + metronidazole: 8(8%)	*p*-value: 0.788	Day 3	Single antibiotic: 6.33; Triple antibiotics: 6.22
Kosus et al., 2010	Superficial and deep	Ceftriaxone + rifamycin SV: 0 out of 596 Ceftriaxone: 12(2%)	*P*-value < 0.05	Day 2, 5, 40	7
**ONCOLOGIC SURGERY**					
Patil et al., 2011	NA	Single antibiotic: 11(47.8%) Combination of antibiotics: 7(25%)	NA	NA	Single antibiotic: 36 Combination of antibiotics: 33
Gulluoglu et al., 2013	Superficial	Ampicillin/sulbactam: 9(4.8%) Non-prophylactic antibiotics: 25(13.7%)	NA	Day 30	NA
El-Mahallawy et al., 2013	NA	Penicillin G sodium + gentamicin: 11(11%) Clindamycin + amikacin: 8(8%)	P value: 0.47	NA	NA

* *The provided percentages were the percentages within the group. CI, confident interval; ICU, intensive care unit; i.v., intravenous; LoS, length of stay; NA, not available; PPSAs, prolonged prophylaxis systemic antimicrobials; SWI, sternal wound infection; T0, No systemic antibiotics + plain cement + conventional ventilation; T1, systemic antibiotics + plain cement + conventional ventilation; T2, non-systemic antibiotics + plain cement + laminar airflow; T3, systemic antibiotics + plain cement + laminar airflow; T4, non-systemic antibiotics + antibiotic-impregnated cement + conventional ventilation; T5, systemic antibiotics + antibiotic-impregnated cement + conventional ventilation; T6, systemic antibiotic + antibiotic-impregnated cement + laminar airflow; T7, systemic antibiotics + antibiotic-impregnated cement + ventilation + body exhaust suit; T8, systemic antibiotics + antibiotic-impregnated cement + laminar ventilation + body exhaust suit*.

### Timing of antibiotic prophylactic interventions

The starting time of antibiotics in prophylactic administrations was different ranging from an hour before the surgical procedure to the time of skin incision. Five studies explicitly stated at the time of starting the prophylactic antibiotics (Chaudhuri et al., 2006; Dhadwal et al., 2007; Wilson et al., 2008; Matsui et al., 2014; Ozdemir et al., 2016). Chaudhuri et al. (2006) and Wilson et al. (2008) reported the administration of the agents 30 min preoperatively for cefuroxime, metronidazole, and cefotetan. Rifampicin in the study conducted by Dhadwal et al. (2007) was administered orally an hour before incision and followed by vancomycin post-induction of anesthesia. Additionally, intravenous cefazolin sodium was injected before skin incision. Elongation of antibiotic prophylaxis was also expounded in the studies, for instance, being explicitly analyzed by Chaudhuri et al. (2006), Dhadwal et al. (2007), and Alekwe et al. (2008).

### Reports of the microorganisms causing SSI

From 7 included studies, this review generated a list of 24 bacteria that were reported as causing SSIs at the site of surgery on the cranium, thorax, abdomen, and thoracolumbar spine (Dhadwal et al., 2007; Kosus et al., 2010; El-Mahallawy et al., 2013; Gulluoglu et al., 2013; Theologis et al., 2014; Lewis et al., 2016; Ozdemir et al., 2016). The predominant species that have been reported to be found for SSIs were gram-negative bacteria. The most common pathogen reported among studies was *Escherichia coli* isolates, accounting for 6.7–50% of incidence in general surgery, orthopedic, cardiothoracic surgery, and cesarean section (Dhadwal et al., 2007; Kosus et al., 2010; Theologis et al., 2014; Ozdemir et al., 2016). More importantly, *S. aureus* was the second most prevalent which was dominant among gram-positives causing SSIs (Dhadwal et al., 2007; El-Mahallawy et al., 2013; Gulluoglu et al., 2013; Ozdemir et al., 2016). Anaerobic bacteria were also reported, with an isolated case of *Bacillus fragilis* as a rare bacteria, accounting for ~13% of the SSI causes among cesarean section procedures (Kosus et al., 2010). We compiled the results of the pattern of bacterial causation of SSIs in Table 5.

**Table 5:** Characteristics of pathogens, prophylactic antibiotic, mean cost and SSI incidence in each surgical procedure.

**Type of surgery, reference**	**Pathogen (%)**	**Prophylactic antibiotic**	Mean cost, US$^*^	**SSI incidence, %**
**General surgery**				
Chaudhuri et al., 2006; Wilson et al., 2008; Matsui et al., 2014; Singh et al., 2014; Ozdemir et al., 2016				
- Colorectal surgery - Excision of pilonidal sinuses - Laparoscopic cholecystectomy	*Escherichia coli* (25) *Klebsiella pneumonia* (50) *Staphylococcus aureus* (25)	Cefazolin Cefotetan Ertapenem Gentamicin Metronidazole Triclosan.	791.59–54,841.32	0.8-71.1
**Orthopedic**				
Elliott et al., 2010; Courville et al., 2012; Merollini et al., 2013; Theologis et al., 2014; Graves et al., 2016; Ceballos et al., 2017				
- Deformity reconstruction - Hip arthroplasty - Hip replacement - Knee arthroplasty - Lower limb amputation	*Citrobacter freundii* (6.7) *Corynebacterium afermentan* (6.7) *Corynebacterium jeikeium* (6.7) *Enterobacter cloacae* (6.7) *Enterobacter cloacae* (6.7) *Escherichia coli* (6.7) MRSA (39.9) *Pseudomonas mirabilis* (13.3) *Staphylococcus epidermidis* (6.7)	Cefazolin Cefotaxime Cefoxitin Cefuroxime Cephalotin Mupirocin Vancomycin	1,245.83–120,989.52	0.5-38.3
**Neurosurgery**				
Emohare et al., 2014; Lewis et al., 2016				
- Cranial surgery - Posterior spinal surgery - Subdural and subgaleal drains	*Enterobacteriacea* (33.3) *Klebsiella pneumonia* (33.3) acnes (33.3)	Cefazolin Vancomycin	887.79–2,879.02	0-3.4
**Cardiothoracic surgery**				
Dhadwal et al., 2007; Joshi et al., 2016				
- Cardiac surgery - Coronary artery bypass	*Staphylococcus aureus* (8.7) *Bacteroides fragilis* (4.3) *Enterobacter cloacae* (2.9) *Enterobacteriaceae* (30.4) *Enterococcus faecalis* (14.5) *Escherichia coli* (24.6) *Klebsiella pneumonia* (3.0) *Pseudomonas aeruginosa* (7.2) *Proteus mirabilis* (3.0) *Serratia marcescens* (1.4)	Gentamicin Rifampicin Vancomycin	7,835.59–22,130.53	1.4-25.3
**Obstetric gynecological surgery**				
Alekwe et al., 2008; Kosus et al., 2010				
- Cesarean section	*Bacteroides (Bacillus) fragilis* (12.5) *Escherichia coli* (50) *Enterococci* (25) *Streptococci spp.s Group B* (12.5)	Ampicillin Ceftriaxone Gentamicin Metronidazole Rifamycin	482.59	0-8
**Oncologic surgery**				
Patil et al., 2011; El-Mahallawy et al., 2013; Gulluoglu et al., 2013				
- Bladder cancer surgery - Breast cancer surgery - Head and neck onco-surgeries	*Acinectobacter haemolyticus* (2.7) *Staphylococcus aureus* (32.4) *Streptococci* (16.2) *Staphylococcus epidermidis* (35.1)	Amikacin Ampicillin Cefazolin Cefprozil	Not adequately informed	4.8-47.8
- Rectal cancer surgery - Stomach cancer surgery	Various gram negatives (13.6)	Ciprofloxacin Clindamycin Gentamicin Metronidazole Moxifloxacin Penicillin G		

* *Adjusted mean cost in US$ at 2015-inflation rate*.

*MRSA, Methicillin-resistance Staphylococcus aureus*.

### Quality assessments of the included studies

The range of CHEC scores in the included studies was from a low of 8 to a high of 18.5 (Dhadwal et al., 2007; Graves et al., 2016). The quality assessment scores of studies regarding general surgery ranged from 10 to 12 (Chaudhuri et al., 2006; Wilson et al., 2008; Matsui et al., 2014; Singh et al., 2014; Ozdemir et al., 2016). Among studies on orthopedic surgery and neurosurgery, the quality ranged between 12 and 18.5 (Elliott et al., 2010; Courville et al., 2012; Merollini et al., 2013; Emohare et al., 2014; Theologis et al., 2014; Graves et al., 2016; Lewis et al., 2016; Ceballos et al., 2017). Two cardiothoracic studies scored 8 and 11.5 points for CHEC items (Dhadwal et al., 2007; Joshi et al., 2016). Two obstetric and gynecological studies were scored at 10.5 and 11 (Alekwe et al., 2008; Kosus et al., 2010). Furthermore, two oncologic surgery studies obtained quality scores of 9.5 and 12.5 (Patil et al., 2011; El-Mahallawy et al., 2013; Gulluoglu et al., 2013). From the CHEC items, concerns mostly related to incremental analysis and sensitivity analysis. The quality assessments of each article are reported in Table 6.

**Table 6:** Quality assessment of each individual study according to Consensus on Health Economic Criteria (CHEC).

**No**.	**Items**	**Chaudhuri et al**	**Wilson et al**	**Matsui et al**	**Singh et al**	**Ozdemir et al**	**Elliott et al**	**Courville et al**	**Merollini et al**	**Theologis et al**	**Graves et al**	**Ceballos et al**	**Emohare et al**	**Lewis et al**	**Dhadwal et al**	**Joshi et al**	**Alekwe et al**	**Kosus et al**	**Patil et al**	**Gulluoglu et al**	**El-Mahallawy et al**
1	Is the study population clearly described?	Y	Y	Y	Y	Y	Y	Y	Y	Y	Y	Y	Y	Y	Y	Y	Y	Y	Y	Y	Y
2	Are competing alternatives clearly described?	Y	Y	Y	Y	Y	Y	Y	Y	Y	Y	Y	Y	Y	Y	Y	Y	Y	Y	Y	Y
3	Is a well-defined research question posed in answerable form?	N	Y	Y	Y	Y	Y	Y	Y	Y	Y	Y	Y	Y	N	Y	N	Y	Y	Y	Y
4	Is the economic study design appropriate to the stated objective?	Y	Y	Y	Y	Y	Y	Y	Y	Y	Y	Y	Y	Y	Y	Y	Y	Y	Y	Y	Y
5	Is the chosen time horizon appropriate to include relevant costs and consequences?	N	N	N	Y	Y	Y	Y	Y	Y	Y	Y	Y	Y	N	Y	N	N	Y	N	N
6	Is the actual perspective chosen appropriate?	N	N	N	Y	N	Y	Y	Y	N	Y	Y	N	N	N	N	N	N	N	N	N
7	Are all important and relevant costs for each alternative identified?	Y	N	Y	Y	N	Y	Y	Y	Y	Y	Y	Y	Y	N	Y	N	Y	N	N	Y
8	Are all costs measured appropriately in physical units?	U	Y	Y	Y	N	Y	Y	Y	Y	Y	Y	U	Y	U	Y	U	Y	N	N	Y
9	Are costs valued appropriately?	U	U	U	Y	N	Y	Y	Y	U	Y	Y	U	U	U	U	U	U	U	U	U
10	Are all important and relevant outcomes for each alternative identified?	Y	Y	Y	Y	Y	Y	Y	Y	Y	Y	Y	Y	Y	Y	Y	Y	Y	Y	Y	Y
11	Are all outcomes measured appropriately?	Y	Y	Y	Y	Y	Y	Y	Y	Y	Y	Y	Y	Y	Y	Y	Y	Y	Y	Y	Y
12	Are outcomes valued appropriately?	U	U	U	Y	Y	U	Y	U	U	U	Y	U	U	U	U	U	U	U	U	Y
13	Is an incremental analysis of costs and outcomes of alternatives performed?	N	N	N	U	N	Y	U	Y	N	Y	Y	N	N	N	N	N	N	N	N	N
14	Are all future costs and outcomes discounted appropriately?	U	U	Y	Y	N	Y	Y	Y	U	Y	Y	U	U	U	U	U	U	U	U	N
15	Are all important variables, whose values are uncertain, appropriately subjected to sensitivity analysis?	N	N	N	Y	N	U	Y	Y	N	Y	Y	N	N	N	N	N	N	N	N	N
16	Do the conclusions follow from the data reported?	Y	Y	N	Y	Y	Y	Y	Y	Y	Y	Y	Y	Y	N	Y	Y	Y	Y	Y	Y
17	Does the study discuss the generalizability of the results to other settings and patient/client groups?	N	Y	Y	Y	Y	Y	Y	Y	Y	Y	Y	N	Y	N	N	Y	N	N	N	Y
18	Does the article indicate that there is no potential conflict of interest of study researcher(s) and funder(s)?	N	N	N	Y	Y	N	Y	Y	N	Y	U	Y	Y	Y	N	Y	N	Y	N	N
19	Are ethical and distributional issues discussed appropriately?	Y	N	Y	N	Y	N	N	N	N	Y	N	N	N	N	N	Y	N	N	Y	Y
Total score		10	10.5	12	17.5	12	16	17.5	17.5	12.5	18.5	17.5	12	13.5	8	11.5	11	10.5	10.5	9.5	12.5

*N,no, with no points; U,Unclear, unclear with half a point; Y,Yes, with one point*.

## Discussion

Guidance for the reporting of economic and clinical studies in the specific field of infectious disease and antibiotic use is urgently needed. Choosing the use of prophylactic antibiotics especially for SSIs should take into account the local epidemiological data of the pathogens and antimicrobial susceptibility. The microbial etiology of SSIs and antibiotic resistance are completely missing from reports of the mid-and long-term economic impact of antibiotic use. For the economic part, in general, the minimum requirements of the established CHEC checklist can assist in the reporting of economic studies. On the other hand, the different checklist from the Consolidated Health Economic Evaluation Reporting Standards (CHEERS) statement has been performed to assess the quality of economic study (Evers et al., 2005; Husereau et al., 2013). However, the CHEERS checklist only considers the completeness of reporting and does not evaluate the quality. The other checklist which was developed by Caro et al. (2012) was merely to conceptualize for model-based studies. Hence, the CHEC checklist is more applicable and appropriate for quality assessment in this review.

The diversity in definition of SSIs in terms of the period to identify the SSIs potentially generates under-reporting of the diseases' occurrence. Despite the definition from the CDC (Horan et al., 1992), other definitions were addressed by Peel and Taylor (1991) from the Surgical Infections Society Study Group (SIGS) and Ayliffe et al. (1993) from the National Prevalence Survey (NPS) which considered grouping wound infection based on the cause of infection, the time of appearance, and the severity of infection. For the time of the appearance of infection, they divided this into three categories, namely early, intermediate, and late based on whether the infection appeared in a 30-day period, in a period of between 1 and 3 months, and over 3 months post-surgery, respectively. By these definitions, the reimbursement for the cost be accurately predicted especially in the extensive financial evaluation of late-occurrence SSIs. Twelve included studies (60%) defined the time for the appearance of SSIs within diverse follow-up intervals for trial-based studies and time horizons for model-based studies.

Obviously, the clinical outcome depends not only on prophylactic antibiotics which is prior to surgical procedures but also on minimal intervention comprising limited tissue damage, which has the effect of accelerating wound healing (Khodakaram et al., 2016). Therefore, the other influential issues that were identified as high-cost in the management of surgical patients include surgical techniques, skilled surgeons, types of diseases, and the for-profit or not-profit nature of healthcare system services (Ogola and Shafi, 2016; Leaper and Edmiston, 2017). The desired economic impacts of the proper use of prophylactic antibiotics in SSIs prevention are shorter lengths of stay, lower resistance rates, and ultimately, the reduction of costs. Some evidence showed a positive relationship between the infection rate and length of stay, and the reason given was that inpatients are at a high risk of infection by nosocomial and often antibiotic- and multi-resistant microorganisms (Pereira et al., 2015; Al-Mousa et al., 2016; Maseda et al., 2016; Karanika et al., 2017; Salgado Yepez et al., 2017). Costs for a day of hospital stay and re-hospitalizations especially in the short-term are virtually fully fixed (Roberts et al., 2009). For instance, a prospective study with a hospital perspective included direct medical costs by calculation based on length of hospital stay in nosocomial infections after head and neck cancer surgery (Penel et al., 2008). Of the included studies 9(45%) included length of hospitalization in their evaluation. Moreover, costs due to antimicrobial resistance indicate the secondary costs for advanced medications to overcome the resistance rates can be expected to eventually become variable costs (Roberts et al., 2009). Timing for administration of prophylactic antibiotics is essential to evaluate the clinical effectiveness, resistance, and costs. A previous RCT in London stated that administration of prophylactic antibiotics within 2 h prior to incision had the lowest risk of SSIs (Classen et al., 1992). Regarding the frequency of the drug administration, an included study showed that prolonged prophylactic use after 24 h post-surgery did not show any benefit in cost and SSI prevention (Lewis et al., 2016).

With regards to new antibiotics, the pricing process has a significant influence on the calculation of the economic outcomes, and thus bias potentially comes particularly from trial-based economic studies that are sponsored by the pharmaceutical industry. The industry can affect the way in which results are reported (Bell et al., 2006; John-Baptiste and Bell, 2010). Disclosure of either funding contributions or conflicts of interest in all the works and the findings of each study is a recommended strategy to identify potential bias (Palumbo et al., 2004). Half of the included studies explicitly included a statement of conflict of interest. It is essential to adjust the costs for antibiotics especially for patented drugs that could decrease significantly in price when the patent period expires. Only 7(35%) included studies reported the costs of the antibiotics including the price of a single dose.

In the economic evaluation, the outcome parameters are holistic including costs, clinical effectiveness, and utility. Hence, a narrow or restricted perspective fosters omission of some essential costs and outcomes. Half of the included studies did not explicitly state the perspective, hence here may be cost measurement omission bias (Evers et al., 2005). For both trial and model-based studies, the societal perspective has a broader view and its use is recommended in economic evaluation (Jonsson, 2009). The included study by Singh et al. (2014) showed that the societal perspective had a 10 times higher cost compared with healthcare and payer perspectives since not only direct costs but also productivity loss is considered comprehensively in a societal perspective which is also referred to as a patient's perspective (Drummond et al., 2015). Of the included studies only 3(15%) took into account the societal perspective with DALY and QALY as utility units. Most of the included studies (75%) performed CMA as the method to analyze the costs for SSIs. Obviously, CMA was simply used and implemented to address the costs due to the presence of SSIs such as in two studies in cesarean section and orthopedics which reported the median cost for SSIs at US$4,091 and US$108,782, respectively (Olsen et al., 2008; Thakore et al., 2015). The values were in line with the findings from the included studies which amounted to between US$482 and US$120,989. The high burden of post-surgical procedures when SSIs are concomitantly present with nosocomial pneumonia is also a complication post-surgery. The additional direct medical cost was considered to increase from EUR19,000 for SSIs to EUR35,000 for both post-surgical complications (Penel et al., 2008). Furthermore, in clinical outcome measurements, there is some evidence that systemic prophylactic antibiotics have a significant impact on minimizing the incidence of SSIs and medical costs in high-risk patients, especially in major surgical procedures including oncologic surgery (Jones et al., 2014), cardiothoracic (Lador et al., 2012), cesarean section (Smaill and Grivell, 2014), and orthopedic surgery (Brown et al., 2004). To achieve high efficacy, a current strategy is a prophylactic combination added locally to the standard prophylaxis, especially in deep surgical sites, for instance, using intra-wound vancomycin (Xiong et al., 2014) or gentamicin (Friberg et al., 2005). A meta-analysis showed that implantable gentamicin-collagen reduced either superficial or deep wound infection effectively, even though the mortality rate was not significantly different (Kowalewski et al., 2015). The use of a local or intra-wound antibiotic as an add-on treatment can be predicted as more effective since the site-target concentration of antibiotics with local treatment is higher than that without local antibiotics. In contrast, Eklund et al. (2005) stated that there was no statistically significant difference in SSI rates between an add-on local gentamicin group and the group without local prophylaxis.

The scope of a cost analysis is critical when evaluating the relevant costs and the patient's expectations on clinical outcomes and safety. To achieve successful treatments especially in the use of antibiotics, antimicrobial susceptibility and the pattern of pathogens causing SSIs should be taken into account. Under-reported unsusceptible antibiotics in the group of high SSI rates can potentially produce bias especially in the interpretation of the treatment outcomes. Therefore, failure in clinical improvement from surgical wounds should consider the local epidemiology susceptibility of antibiotics. None of the included studies reported on antimicrobial susceptibility. Notably, regarding SSIs the importance of correct and early diagnosis cannot be stressed enough. Here, microbiological diagnostics are paramount in decisions for specific antibiotic treatment. Treating infection in the most effective method with the correct antibiotics is important with respect to the treatment, but also with respect to the development of antibiotic resistance (Dik et al., 2015). An integrated stewardship program, such as the AID stewardship (Antibiotic, Infection Prevention, and Diagnostic Stewardship) is crucial since it targets all different aspects of infection management. This theragnostic approach involves a combination between diagnostics and therapeutics considering the interdisciplinary staff in the complexity of infection management. The role of diagnostic stewardship is especially gaining momentum right now to achieve a personalized approach in infection management (Dik J. H. et al., 2017; Dik J.-W. H. et al., 2017; Messacar et al., 2017). Therefore, this review comprehensively takes into account all stewardship aspects on each surgical procedure in terms of the effectiveness of prophylactic therapeutics, diagnostics to determine SSIs the pathogens, patient safety, antibiotic resistance, timing of prophylaxis, and further impact on costs.

We are aware that this review has limitations. Notably, the study may be less representative of other important procedures such as urological, ophthalmological, organ transplantations, implantable devices, and dental surgery. Nearly 15% of 441 patients undergoing kidney transplantation in a hospital in the US developed SSIs. In the 2013 annual report, almost 18,000 patients have carried out kidney transplantation procedures (National Institutes of Health, 2015). It indicated a high number of potential SSIs coming from the procedures and obviously a need exists to perform an analysis on the cost and effectiveness of the use of prophylactic antibiotics. Of 208 eligible studies, only 3 studies referred to SSIs in urology; nevertheless these studies did not meet the further inclusion criteria. Using different definitions to determine the infections potentially leads to underreporting of SSIs and the location or types of SSIs, even in community health services. Of the included studies, the only one reported incidence of the SSIs based on the types (Emohare et al., 2014). The reporting of updated data related to microbiological results is fruitful, even though it may be more difficult to determine the definite cause of SSI at particular sites of the incision from the results. In the study, none of the included studies considered procedures in children or pediatric surgery which has a higher risk of SSIs and different pathogen patterns. Moreover, because of major differences in the incidence of antibiotic resistance between the US and Europe, outcome studies need to be interpreted with caution. Finally, this review used the CHEC as a rigorous method to assess the quality of the articles and can be used as a baseline for guidelines for further economic evaluations (Evers et al., 2015). During the work of this review, we have followed a standard checklist from PRISMA in reporting systematic review. The checklist is shown in Table S4.

## Conclusions

Overall, we describe novel findings from reviewing the economic evaluations of studies concerning prophylactic antibiotic uses for SSI prevention in general surgery, orthopedic surgery, neurosurgery, cardiothoracic surgery, obstetric and gynecological surgery, and oncologic surgery. Preoperative prophylactic antibiotics administered either locally or systemically are considered in some studies and for specific interventions at preventing SSIs. The quality in reporting of economic evaluation indicates that the included studies need to be improved for further research, especially with respect to issues related to antimicrobial susceptibility, pathogens causing SSIs, cost perspectives, incremental analysis, and sensitivity analysis of the costs. Notably, the valuable information in terms of cost, updated causes of SSIs from this review can be considered in the clinical implementation in the proper use of prophylactic antibiotics to reduce costs and to prevent SSIs and further antimicrobial resistance.

## Author contributions

AP, MP, J-WD, and AF initially contributed to develop the concept and the design of the study and wrote the initial manuscript. AP, DS, and J-WD identified the eligible and selected studies from PubMed and Embase. AP and J-WD performed data analysis and synthesis. AP and DS conducted the quality assessment and all authors revised the work and approved the final draft before submission.

### Conflict of interest statement

MP received grants and honoraria from various pharmaceutical companies, all unrelated to this research except one recent Advisory Board (Pfizer) on the *Staphylococcus aureus* vaccine to prevent SSI. The remaining authors declare that the research was conducted in the absence of any commercial or financial relationships that could be construed as a potential conflict of interest.
