# Supplementary material for: Prevention of Surgical Site Infections: A Systematic Review of Cost Analyses in the Use of Prophylactic Antibiotics
Source: Front Pharmacol. 2018 Jul 18;9:776. doi: 10.3389/fphar.2018.00776 (PMC6060435; doi:10.3389/fphar.2018.00776)
Supplement: Supplementary file 1 [file Table_1.DOCX]

Supplementary Material

Prevention of Surgical Site Infections: A Systematic Review of Cost Analyses in the Use of Prophylactic Antibiotics

Abdul K.R. Purba^1,2,3*^, Didik Setiawan^4,5^, Erik Bathoorn^3^, Maarten J. Postma^1,2,4,6^, Jan-Willem Dik^3^, Alex W. Friedrich^3^

^1^Department of Health Sciences, University of Groningen, University Medical Center Groningen, Groningen, Netherlands

^2^Department of Pharmacology and Therapy, Universitas Airlangga, Faculty of Medicine, Surabaya, Indonesia

^3^University of Groningen, University Medical Center Groningen, Department of Medical Microbiology, Groningen, Netherlands

^4^Unit of PharmacoEpidemiology & Pharmacoeconomics (PE2), University of Groningen, Department of Pharmacy, Groningen, Netherlands.

^5^Department of Pharmacology and Clinical Pharmacy, Faculty of Pharmacy, Universitas Muhammadiyah Purwokerto, Purwokerto, Indonesia

^6^Department of Economics, Econometrics & Finance, University of Groningen, Faculty of Economics & Business, Groningen, Netherlands

*** Correspondence:**Abdul Khairul Rizki Purba

Email: [khairul_purba@fk.unair.ac.id](mailto:khairul_purba@fk.unair.ac.id)

# Supplementary Tables

**Table S1. Search strategy using PubMed database**

| PICO | Strategy strings | The number of articles |
| --- | --- | --- |
| P | "Surgical Procedures, Operative"[Mesh]) OR surger*[tiab] OR surgical[tiab] OR operation*[tiab] OR operative*[tiab] | 3,783,815 |
| I | "Anti-Infective Agents"[Mesh] OR "Anti-Infective Agents" [Pharmacological Action] OR "Antibiotic Prophylaxis"[Mesh] OR antimicrob*[tiab] OR antibiotic*[tiab] | 1,675,192 |
|  |  |  |
| O1 | "Surgical Wound Infection"[Mesh]OR surgical site infection*[tiab] OR surgical wound infection*[tiab] OR SSI*[tiab] | 39,143 |
| O2 | "Costs and Cost Analysis"[Mesh] OR cost*[tiab] OR econom*[tiab] OR financ*[tiab] OR Pharmacoeconomic*[tiab] | 810,532 |
| P+I+C+O1+O2 | (“Surgical Procedures, Operative”[Mesh]) OR surger*[tiab] OR surgical[tiab] OR operation*[tiab] OR operative*[tiab]) AND (“Anti-Infective Agents”[Mesh] OR “Anti-Infective Agents” [Pharmacological Action] OR “Antibiotic Prophylaxis”[Mesh] OR antimicrob*[tiab] OR antibiotic*[tiab]) AND ("Surgical Wound Infection"[Mesh]OR surgical site infection*[tiab] OR surgical wound infection*[tiab] OR SSI*[tiab]) AND ("Costs and Cost Analysis"[Mesh] OR cost*[tiab] OR econom*[tiab] OR financ*[tiab] OR Pharmacoeconomic*[tiab]) | 1,079 |
| Filter | From 1 January 2006 to 31 August 2017 | 644 |
