# Supplementary material for: Prevention of Surgical Site Infections: A Systematic Review of Cost Analyses in the Use of Prophylactic Antibiotics
Source: Front Pharmacol. 2018 Jul 18;9:776. doi: 10.3389/fphar.2018.00776 (PMC6060435; doi:10.3389/fphar.2018.00776)
Supplement: Supplementary file 2 [file Table_2.DOCX]

Supplementary Material

Prevention of Surgical Site Infections: A Systematic Review of Cost Analyses in the Use of Prophylactic Antibiotics

Abdul K.R. Purba^1,2,3*^, Didik Setiawan^4,5^, Erik Bathoorn^3^, Maarten J. Postma^1,2,4,6^, Jan-Willem Dik^3^, Alex W. Friedrich^3^

^1^Department of Health Sciences, University of Groningen, University Medical Center Groningen, Groningen, Netherlands

^2^Department of Pharmacology and Therapy, Universitas Airlangga, Faculty of Medicine, Surabaya, Indonesia

^3^University of Groningen, University Medical Center Groningen, Department of Medical Microbiology, Groningen, Netherlands

^4^Unit of PharmacoEpidemiology & Pharmacoeconomics (PE2), University of Groningen, Department of Pharmacy, Groningen, Netherlands.

^5^Department of Pharmacology and Clinical Pharmacy, Faculty of Pharmacy, Universitas Muhammadiyah Purwokerto, Purwokerto, Indonesia

^6^Department of Economics, Econometrics & Finance, University of Groningen, Faculty of Economics & Business, Groningen, Netherlands

*** Correspondence:**Abdul Khairul Rizki Purba

Email: [khairul_purba@fk.unair.ac.id](mailto:khairul_purba@fk.unair.ac.id)

# Supplementary Tables

**Table S2. Search strategy using EMBASE database**

| PICO | Strategy strings | The number of articles |
| --- | --- | --- |
| P | 'surgical patient'/exp OR 'surgical patient' OR 'surgery'/exp OR 'surgery' OR surger*:ab,ti OR surgical:ab,ti OR operation*:ab,ti OR operative*:ab,ti | 6,024,616 |
| I | 'antibiotic prophylaxis'/exp OR 'antiinfective agent'/exp OR 'antibiotic agent'/exp OR antimicrob*:ab,ti OR antibiotic*:ab,ti | 2,877,127 |
|  |  |  |
| O1 | 'surgical infection'/exp OR 'surgical site infection*':ab,ti OR 'surgical wound infection*':ab,ti OR ssi*:ab,ti | 44,898 |
| O2 | 'cost effectiveness analysis'/exp OR 'cost'/exp OR 'pharmacoeconomics'/exp OR 'economic evaluation'/exp OR cost*:ab,ti OR econom*:ab,ti OR financ*:ab,ti OR pharmacoeconomic*:ab,ti | 1,228,677 |
| P+I+C+O1+O2 | ('surgical patient'/exp OR 'surgery'/exp OR (surger* OR surgical OR operation* OR operative*):ab,ti) AND ('antibiotic prophylaxis'/exp OR 'antiinfective agent'/exp OR 'antibiotic agent'/exp OR (antimicrob* OR antibiotic*):ab,ti) AND ('surgical infection'/exp OR 'surgical site infection*':ab,ti OR 'surgical wound infection*':ab,ti OR ssi*:ab,ti) AND ('cost effectiveness analysis'/exp OR 'cost'/exp OR 'pharmacoeconomics'/exp OR 'economic evaluation'/exp OR cost*:ab,ti OR econom*:ab,ti OR financ*:ab,ti OR pharmacoeconomic*:ab,ti) | 1,750 |
| Filter | From 1 January 2006 to 31 August 2017 | 1,417 |
