# Supplementary material for: Prevention of Surgical Site Infections: A Systematic Review of Cost Analyses in the Use of Prophylactic Antibiotics
Source: Front Pharmacol. 2018 Jul 18;9:776. doi: 10.3389/fphar.2018.00776 (PMC6060435; doi:10.3389/fphar.2018.00776)
Supplement: Supplementary file 3 [file Table_3.DOCX]

Supplementary Material

Prevention of Surgical Site Infections: A Systematic Review of Cost Analyses in the Use of Prophylactic Antibiotics

Abdul K.R. Purba^1,2,3*^, Didik Setiawan^4,5^, Erik Bathoorn^3^, Maarten J. Postma^1,2,4,6^, Jan-Willem Dik^3^, Alex W. Friedrich^3^

^1^Department of Health Sciences, University of Groningen, University Medical Center Groningen, Groningen, Netherlands

^2^Department of Pharmacology and Therapy, Universitas Airlangga, Faculty of Medicine, Surabaya, Indonesia

^3^University of Groningen, University Medical Center Groningen, Department of Medical Microbiology, Groningen, Netherlands

^4^Unit of PharmacoEpidemiology & Pharmacoeconomics (PE2), University of Groningen, Department of Pharmacy, Groningen, Netherlands.

^5^Department of Pharmacology and Clinical Pharmacy, Faculty of Pharmacy, Universitas Muhammadiyah Purwokerto, Purwokerto, Indonesia

^6^Department of Economics, Econometrics & Finance, University of Groningen, Faculty of Economics & Business, Groningen, Netherlands

*** Correspondence:**Abdul Khairul Rizki Purba

Email: [khairul_purba@fk.unair.ac.id](mailto:khairul_purba@fk.unair.ac.id)

# Supplementary Tables

**Table S3. Assessment of eligible studies**

| **No** | **Title** | **Authors** | **Countries** | **Surgical type** | **Economic evaluation** | **Assessment** |
| --- | --- | --- | --- | --- | --- | --- |
| 1 | A budget impact analysis of three presurgical skin antisepsis protocols | Zhou et al., 2010 | The US | NS | CEA | No analysis on prophylactic antibiotic |
| 2 | A bundle approach for cesarean section surgical site infection prevention results in a zero percent surgical site infection rate in C-section patients | Zajac et al., 2011 | The US | Obstetric gynecology | CMA | No analysis on prophylactic antibiotic |
| 3 | A cost-effectiveness modelling study of strategies to reduce risk of infection following primary hip replacement based on a systematic review. | Graves et al., 2016 | The US | Orthopedic | CUA | Included |
| 4 | A journey to zero: Reduction of post-operative cesarean surgical site infections over a five-year period. | Hickson et al. 2015 | The US | Obstetric gynecology | CMA | No analysis on prophylactic antibiotic |
| 5 | A multifaceted approach reduces surgical site infection rates, incidents, and associated costs for abdominal hysterectomy and caesarean section patients | Mauzey., 2012 | The US | Obstetric gynecology | CMA | No analysis on prophylactic antibiotic |
| 6 | A nursing intervention to reduce pediatric sternal surgical site infections (SSIs). | Walbay et al. 2011 | The US | Cardiothoracic surgery | CMA | Review |
| 7 | A prospective study of outcomes, healthcare resource utilization, and costs associated with postoperative nosocomial infections. | Herwaldt et al. 2006 | The US | NS | CMA | No analysis on prophylactic antibiotic |
| 8 | A standardized protocol for perioperative antibiotic prophylaxis is associated with improvement of timing and reduction of costs. | Willemsen et al., 2007. | The Netherlands | NS | CMA | No analysis on prophylactic antibiotic |
| 9 | A systematic review and economic model of switching from non-glycopeptide to glycopeptide antibiotic prophylaxis for surgery. | Cranny et al., 2008. | The UK | NS | CMA | Review |
| 10 | Achieving and Sustaining Zero: Preventing Surgical Site Infections After Isolated Coronary Artery Bypass With Saphenous Vein Harvest Site Through Implementation of a Staff-Driven Quality Improvement Process. | Kles et al. 2015 | The US | Cardiothoracic surgery | CMA | No analysis on prophylactic antibiotic |
| 11 | Additional direct medical costs associated with nosocomial infections after head and neck cancer surgery: a hospital-perspective analysis. | Penel et al., 2008. | Denmark | Oncosurgery | CMA | No analysis on prophylactic antibiotic |
| 12 | Additional direct medical costs of nosocomial infections: an estimation from a cohort of patients in a French university hospital. | Defez et al., 2008 | French | NS | CMA | No analysis on prophylactic antibiotic |
| 13 | Adherence with guidelines of perioperative antibiotic prophylaxis and cost among women undergoing cesarean section. | Apostolopoulou et al., 2010. | Greece | Obstetric gynecology | CMA | No analysis on prophylactic antibiotic |
| 14 | Adverse Clinical Outcomes and Resource Utilization Associated with Methicillin-Resistant and Methicillin-Sensitive Staphylococcus aureus Infections after Elective Surgery. | Campbell et al., 2015. | The US | NS | CMA | No analysis on prophylactic antibiotic |
| 15 | An economic model for the prevention of MRSA infections after surgery: non-glycopeptide or glycopeptide antibiotic prophylaxis?. | Elliott et al., 2010. | Germany | Orthopedic | CUA | Included |
| 16 | An economic model: value of antimicrobial-coated sutures to society, hospitals, and third-party payers in preventing abdominal surgical site infections. | Singh et al 2014 | The US | General Surgery | CEA | Included |
| 17 | Antibiotic prophylaxis for subdural and subgaleal drains. | Lewis et al., 2017 | The US | Neurosurgery | CMA | Included |
| 18 | Antibiotic prophylaxis for surgery for proximal femoral and other closed long bone fractures. | Gillespie et al., 2010 | England | Orthopedic | CMA | Review |
| 19 | Antibiotic prophylaxis in laparoscopic cholecystectomy: a randomized controlled trial. | Matsui et al., 2014 | The US | General surgery | CMA | Included |
| 20 | Antibiotic-Loaded Cement Articulating Spacer for 2-Stage Reimplantation in Infected Total Knee Arthroplasty. A Simple and Economic Method | Hsu et al., 2007 | Hong kong | Orthopedic | CMA | Review |
| 21 | Antimicrobial prophylaxis for surgical site infections with increasing antibiotic resistance? | Metodiev, 2013 | Bulgaria | NS | CMA | Review |
| 22 | Application of ATC/DDD methodology to evaluate perioperative antimicrobial prophylaxis | Akalin et al., 2012 | Turkey | General surgery | CMA | No analysis on SSI outcomes |
| 23 | Are lower levels of red blood cell transfusion more cost-effective than liberal levels after cardiac surgery? Findings from the TITRe2 randomised controlled trial | Stokes et al., 2016 | The UK | Cardiothoracic surgery | CMA | No analysis on prophylactic antibiotic |
| 24 | Assessment of costs associated with hospital-acquired infections in a private tertiary care hospital in India | Tiwari et al., 2013 | India | NS | CMA | No analysis on prophylactic antibiotic |
| 25 | Brief primer on clostridium difficile infection costs, reimbursement, and government regulation | Frei et al., 2013 | The US | NS | CMA | No analysis on prophylactic antibiotic |
| 26 | Cardiac surgery and sternal wound complications: Iodine impregnated drape (Ioban® 2) versus standard drape. Insight from a prospective comparative analysis of complications and costs | Micciolo et al., 2011 | Italy | Cardiothoracic surgery | CMA | No analysis on prophylactic antibiotic |
| 27 | Centers for Disease Control and Prevention Guideline for the Prevention of Surgical Site Infection, 2017. | Berrios-Torres et al., 2017 | The US | NS | CMA | Review |
| 28 | Challenges in the prevention of surgical site infections | Anaya et al., 2006 | The US | NS | CMA | Review |
| 29 | Chasing zero: The drive to eliminate surgical site infections | Thompson et al., 2011 | The US | NS | CMA | No analysis on prophylactic antibiotic |
| 30 | Clinical and economic outcomes of pharmacist-managed antimicrobial prophylaxis in surgical patients. | Bond et al., 2007 | The US | NS | CMA | No analysis on prophylactic antibiotic |
| 31 | Clinical and financial outcomes due to methicillin resistant staphylococcus aureus surgical site infection: A multi-center matched outcomes study | Anderson et al., 2009 | The US | NS | CMA | No analysis on prophylactic antibiotic |
| 32 | Clinical pathway of chest surgery for malignant lung tumor - Standard treatment and diagnosis procedure combination | Ito et al., 2009 | Japan | Oncosurgery | CMA | No analysis on prophylactic antibiotic |
| 33 | Commentary on: Does Implant Insertion with a Funnel Decrease Capsular Contracture? A Preliminary Report | Deva., 2016 | Australia | General surgery | CEA | No analysis on prophylactic antibiotic |
| 34 | Comparative analysis of surgical site infection after minimally invasive versus open posterior/transforaminal lumbar interbody fusion: Analysis of hospital billing and discharge data from 5328 patients | McGirt et al., 2010 | The US | Neurosurgery | CMA | No analysis on prophylactic antibiotic |
| 35 | Comparative costs of ertapenem and cefotetan as prophylaxis for elective colorectal surgery. | Wilson et al., 2008 | The US | General surgery | CMA | Included |
| 36 | Comparative effectiveness and cost-benefit analysis of topical vancomycin powder in posterior spinal fusion for spine trauma and degenerative spine disease | Dewan et al., 2013 | The US | Orthopedic | CBA | Review |
| 37 | Comparative effectiveness of pressurized irrigation or subcutaneous antibiotics versus standard wound closure on surgical site infections following colorectal surgery | Dineen et al., 2013 | The US | General surgery | CEA | No analysis on prophylactic antibiotic |
| 38 | Comparing a combination of penicillin G and gentamicin to a combination of clindamycin and amikacin as prophylactic antibiotic regimens in prevention of clean contaminated wound infections in cancer surgery. | El-Mahallawy et al., 2013 | Egypt | Oncosurgery | CEA | Included |
| 39 | Comparison between one and two days treatment with intravenous cefuroxime in laparoscopic cholecystectomy | Khan et al., 2014 | Pakistan | General surgery | CMA | Incomplete data related cost |
| 40 | Comparison of ceftriaxone versus triple drug regimen in the prevention of cesarean section infectious morbidities | Alekwe et al., 2008 | Nigeria | Obstetric gynecology | CMA | Included |
| 41 | Comparison of clinical and economic outcomes of two antibiotic prophylaxis regimens for sternal wound infection in high-risk patients following coronary artery bypass grafting surgery: a prospective randomised double-blind controlled trial. | Dhadwal et al., 2007 | The UK | Cardiothoracic surgery | CMA | Included |
| 42 | Comparison of combinations of ciprofloxacin-metronidazole and ceftriaxone-metronidazole in controlling operative site infections in obstetrics and gynecological surgeries: A retrospective study | Anad et al., 2011 | India | Obstetric gynecology | CMA | Incomplete data related cost |
| 43 | Comparison of control strategies for methicillin-resistant Staphylococcus aureus | Bessesen et al., 2013 | The US | NS | CMA | Lack of information on prophylactic antibiotics |
| 44 | Comparison of Efficacy and Cost of Iodine Impregnated Drape vs. Standard Drape in Cardiac Surgery: Study in 5100 Patients | Bejko et al., 2015 | Italy | Cardiothoracic surgery | CMA | No analysis on prophylactic antibiotic |
| 45 | Comparison of the cost and outcomes following totally laparoscopic and laparoscopy-assisted distal gastrectomies for gastric cancer: a single-institution comparison | Shinohara et al., 2016 | Japan | Oncosurgery | CMA | No analysis on prophylactic antibiotic |
| 46 | Comparison of the use of single and combined antibiotics for head and neck onco-surgeries: A cost effective analysis | Patil et al., 2011 | India | Oncosurgery | CMA | Included |
| 47 | Consequences of failure of initial antibiotic therapy in complicated skin and skin structure infections in US hospitals, 2000-2009 | Berger et al., 2011 | The US | NS | CMA | No analysis on prophylactic antibiotic |
| 48 | CORR insights®: No infection reduction using chlorhexidine wipes in total joint arthroplasty | Della Valle | The US | Orthopedic | CEA | No analysis on prophylactic antibiotic |
| 49 | Cost analysis of antibiotic-impregnated catheters in the treatment of hydrocephalus in adult patients. | Farber et al | The US | Neurosurgery | CEA | No analysis on prophylactic antibiotic |
| 50 | Cost analysis of gentamicin-impregnated collagen sponges in preventing sternal wound infection post cardiac surgery. | Joshi at al., 2016 | The UK | Cardiothoracic surgery | CMA | Included |
| 51 | Cost analysis of surgical site infections | Urban., 2006 | The US | NS | CEA | No analysis on prophylactic antibiotic |
| 52 | Cost and consequences of surgical site infections: A call to arms | Sullivan et al., 2017 | The US | NS | CEA | No analysis on prophylactic antibiotic |
| 53 | Cost benefit analysis of additional doses of antibiotic prophylaxis at the time of ceserean delivery | Crimmins et al., 2012 | The US | Obstetric gynecology | CBA | No analysis on SSI outcomes |
| 54 | Cost burden of inappropriate peri-operative antibacterial use in a teaching tertiary-care rural hospital in India | Shah et al., 2013 | India | NS | CMA | No analysis on prophylactic antibiotic |
| 55 | Cost effectiveness of collagen crosslinking for progressive keratoconus in the UK NHS | Salmon et al., 2015 | The UK | Opthalmic surgery | CUA | No analysis on prophylactic antibiotic |
| 56 | Cost savings analysis of intrawound vancomycin powder in posterior spinal surgery. | Emohare et al., 2014 | The US | Orthopedic | CMA | Included |
| 57 | Cost savings for the use of antibacterial sutures in Spanish hospitals. Budget impact model | Pobre et al., 2014 | Spain | Neurosurgery | CEA | No analysis on prophylactic antibiotic |
| 58 | Cost-benefit analysis of craniocerebral surgical site infection control in tertiary hospitals in China | Zhou et al., 2015 | China | Neurosurgery | CBA | No analysis on prophylactic antibiotic |
| 59 | Cost-Effectiveness Analysis of the Use of a Prophylactic Antibiotic for Patients Undergoing Lower Limb Amputation due to Diabetes or Vascular Illness in Colombia. | Ceballos et al., 2017 | Colombia | Orthopedic | CEA | Included |
| 60 | Cost-effectiveness of a hand hygiene program on health care–associated infections in intensive care patients at a tertiary care hospital in Vietnam | Thi Anh Thu et al., 2015 | Vietnam | Not surgery | CEA | No analysis on prophylactic antibiotic |
| 61 | Cost-effectiveness of a Staphylococcus aureus screening and decolonization program for high-risk orthopedic patients. | Slover et al., 2011 | The US | Orthopedic | CEA | No analysis on prophylactic antibiotic |
| 62 | Cost-effectiveness of linezolid and vancomycin in the treatment of surgical site infections. | Patanwala et al., 2007 | England | NS | CEA | No analysis on prophylactic antibiotic |
| 63 | Cost-effectiveness of perioperative selective decontamination of the digestive tract versus placebo in elective gastrointestinal surgery | Dijksman et al., 2012 | Switzerland | General surgery | CEA | No analysis on prophylactic antibiotic |
| 64 | Cost-effectiveness of preoperative nasal mupirocin treatment in preventing surgical site infection in patients undergoing total hip and knee arthroplasty: a cost-effectiveness analysis. | Courville et al., 2012 | The US | Orthopedic | CEA | Included |
| 65 | Cost-effectiveness of warm humidified CO2 to reduce surgical site infections in laparoscopic colorectal surgery: A cohort study | Mason et al., 2015 | The UK | General surgery | CEA | No analysis on prophylactic antibiotic |
| 66 | Cost-utility analysis of repair of reducible ventral hernia | Stey et al., 2014 | The US | General surgery | CUA | No analysis on prophylactic antibiotic |
| 67 | Costs of surgical site infections in selected procedures in orthopedic surgery | Rozanska et al., 2010 | Poland | Orthopedic | CMA | No analysis on prophylactic antibiotic |
| 68 | Current options in inguinal hernia repair in adult patients | Kulacoglu et al., 2011 | Turkey | General surgery | CMA | No analysis on prophylactic antibiotic |
| 69 | Day case laparoscopic incisional hernia repair is feasible, acceptable, and cost effective | Engledow et al., 2007 | Germany | General surgery | CEA | No analysis on prophylactic antibiotic |
| 70 | Dealing with MRSA decolonisation failures and cancelled surgeries during NHS financial turbulence: A clinico-economic modelling study | Aggarwal et al., 2010 | The UK | Orthopedic | CEA | No analysis on prophylactic antibiotic |
| 71 | Decreased Hospital Costs and Surgical Site Infection Incidence With a Universal Decolonization Protocol in Primary Total Joint Arthroplasty | Stambough et al., 2017 | The US | NS | CEA | No analysis on prophylactic antibiotic |
| 72 | Decreased infection rates following total joint arthroplasty in a large county run teaching hospital: A single surgeon's experience and possible solution | Gottschalk et al., 2014 | The US | Orthopedic | CMA | No analysis on prophylactic antibiotic |
| 73 | Deep and organ/space infections in patients undergoing elective colorectal surgery: incidence and impact on hospital length of stay and costs. | Eagye et al | The US | General surgery | CMA | No analysis on prophylactic antibiotic |
| 74 | Deep wound infection after proximal femoral fracture: consequences and costs | Pollard et al., 2006 | The UK | Orthopedic | CMA | No analysis on prophylactic antibiotic |
| 75 | Diagnosis and management of skin and soft-tissue infections (SSTI): A literature review and consensus statement on behalf of the Italian society of infectious diseases and international society of chemotherapy | Esposito et al., 2011 | Italy | NS | CMA | Review |
| 76 | Direct costs of antibiotic treatment of hospital acquired infections in Polish hospitals | Rozanska et al., 2010 | Poland | NS | CMA | No analysis on prophylactic antibiotic |
| 77 | Direct hemoperfusion with polymyxin B-immobilized fiber column (PMX-DHP) can improve the prognosis and medical expense | Edamoto et al., 2010 | Japan | NS | CMA | No analysis on prophylactic antibiotic |
| 78 | Economic and clinical contributions of an antimicrobial barrier dressing: a strategy for the reduction of surgical site infections. | Leaper et al., 2010 | England | NS | CEA | No analysis on prophylactic antibiotic |
| 79 | Economic and organizational sustainability of a negative-pressure portable device for the prevention of surgical-site complications | Foglia et al., 2017 | Italy | NS | CMA | No analysis on prophylactic antibiotic |
| 80 | Economic aspects of deep sternal wound infections. | Graf et al., 2010 | Germany | Cardiothoracic surgery | CMA | No analysis on prophylactic antibiotic |
| 81 | Economic burden of surgical site infections at a European university hospital. | Weber et al., 2008 | Switzerland | NS | CMA | No analysis on prophylactic antibiotic |
| 82 | Economic Evaluation of Adjunctive Azithromycin Prophylaxis for Cesarean Delivery. | Harper et al., 2017 | The US | Osbtetric gynecology | CEA | No analysis on prophylactic antibiotic |
| 83 | Economic evaluation of appropriate duration of antibiotic prophylaxis for prevention of neurosurgical infections in a middle-income country. | Ulu-Kilic et al., 2015 | Turkey | Neurosurgery | CMA | No analysis on prophylactic antibiotic |
| 84 | Economic evaluation of chlorhexidine cloths on healthcare costs due to surgical site infections following total knee arthroplasty | Kapadia et al., 2013 | The US | Orthopedic | CMA | No analysis on prophylactic antibiotic |
| 85 | Economic evaluation of interventions for prevention of hospital acquired infections: A systematic review | Arefian et al., 2016 | Germany | NS | CMA | Review |
| 86 | Economic impact of surgical site infection post-cabg surgery using multi-institutional hospitalization data | Patkar et al., 2009 | The US | Cardiothoracic surgery | CMA | No analysis on prophylactic antibiotic |
| 87 | Economic impact of treatment for surgical site infections in cases of total knee arthroplasty in a tertiary public hospital in Brazil | Del-Paz et al., 2010 | Brazil | Obstetric gynecology | CMA | No analysis on prophylactic antibiotic |
| 88 | Economic value of using antimicrobial coated sutures for abdominal incisions to prevent surgical site infections | Singh et al., 2014 | The US | General surgery | CEA | Included |
| 89 | Effect and cost of perioperative use of antibiotics in coronary artery bypass grafting: a randomized controlled study. | Zhou et al., 2011 | China | Cardiothoracic surgery | CEA | No analysis on prophylactic antibiotic |
| 90 | Effect of targeted surveillance for control of methicillin-resistant Staphylococcus aureus in a community hospital system | West et al., 2006 | The US | NS | CEA | No analysis on prophylactic antibiotic |
| 91 | Effectiveness of a Hospital-wide Educational Programme for Infection Control to Reduce the Rate of Health-Care Associated Infections and related Sepsis (ALERTS) Study Synopsis | Hagel et al., 2011 | Germany | NS | CMA | No analysis on prophylactic antibiotic |
| 92 | Effects of pharmaceutical counselling on antimicrobial use in surgical wards: Intervention study with historical control group | Grill et al., 2011 | Germany | NS | CMA | No analysis on prophylactic antibiotic |
| 93 | Efficacy of prophylactic antibiotic administration for breast cancer surgery in overweight or obese patients: a randomized controlled trial. | Gulluoglu et al., 2013 | Turkey | Oncosurgery | CMA | Included |
| 94 | Enhanced recovery program versus traditional care in laparoscopic hepatectomy | Liang et al., 2016 | China | General surgery | CMA | No analysis on prophylactic antibiotic |
| 95 | Epidemiological and economic burden of surgical site infections (SSIS) associated with hip arthroplasty | Patel et al., 2014 | Canada | Orthopedic | CMA | Review |
| 96 | Estimated hospital costs associated with preventable health care-associated infections if health care antiseptic products were unavailable | Schmier et al., 2016 | The US | NS | CMA | Review |
| 97 | Financial Impact of Surgical Site Infection after Kidney Transplantation: Implications for Quality Improvement Initiative Design | Ho et al., 2010 | The US | Urology | CMA | No analysis on prophylactic antibiotic |
| 98 | Funding healthcare-associated infection research: A systematic analysis of UK research investments, 1997-2010 | Head et al., 2014 | The UK | NS | CMA | No analysis on prophylactic antibiotic |
| 99 | Global burden of surgical site infections (SSIs) associated with knee arthroplasty | Yu et al., 2014 | The US | Orthopedic | CMA | Review |
| 100 | Harvest limb infections; the secret surgical nightmare and how to prevent them | Bowles., 2014 | The US | Cardiothoracic surgery | CMA | No analysis on prophylactic antibiotic |
| 101 | Health Utility Values Associated with Surgical Site Infection: A Systematic Review | Gheorghe et al., 2015 | The UK | NS | CMA | Review |
| 102 | Hospital-associated costs due to surgical site infection after breast surgery - Invited critique | Olsen., 2008 | The US | Oncosurgery | CMA | No analysis on prophylactic antibiotic |
| 103 | Hospital-in-the-home treatment of surgical infectious diseases: An economic analysis | Mazo et al., 2007 | Spain | NS | CMA | No analysis on prophylactic antibiotic |
| 104 | Ideal timing of surgery for acute uncomplicated appendicitis | Eko et al., 2013 | The US | General surgery | CEA | No analysis on prophylactic antibiotic |
| 105 | Impact of a prevention and control infection program in a tertiary care teaching hospital | Quiros et al., 2013 | Argentina | NOT SURGERY | CMA | No analysis on prophylactic antibiotic |
| 106 | Impact of an antimicrobial-impregnated gauze dressing on surgical site infections including methicillin-resistant Staphylococcus aureus infections. | Mueller et al., 2008 | The US | NS | CMA | No analysis on SSI outcomes |
| 107 | Impact of antibiotic prophylaxis for intrathecal baclofen pump surgery in pediatric patients. | Pan et al., 2015 | The US | Neurosurgery | CMA | No analysis on prophylactic antibiotic |
| 108 | Impact of antibiotic stewardship on perioperative antimicrobial prophylaxis. | Murri et al., 2016 | Italy | NS | CMA | No analysis on prophylactic antibiotic |
| 109 | Impact of surgical site infection after colorectal surgery on hospital stay and medical expenditure in Japan | Kashimura et al., 2012 | Japan | General surgery | CMA | No analysis on prophylactic antibiotic |
| 110 | Impact of surgical site infections on cost of illness and length of stay in a teaching hospital | Wang et al., 2015 | China | NS | CMA | No analysis on prophylactic antibiotic |
| 111 | Impact of using triclosan-antibacterial sutures on incidence of surgical site infection. | Galal et al., 2011 | Egypt | NS | CMA | No analysis on prophylactic antibiotic |
| 112 | Implementation of an infection prevention bundle to reduce surgical site infections and cost following spine surgery | Featherall et al., 2016 | The US | Neurosurgery | CMA | No analysis on prophylactic antibiotic |
| 113 | Implementing 1-dose antibiotic prophylaxis for prevention of surgical site infection. | Fonseca et al., 2006 | Brazil | NS | CMA | No analysis on prophylactic antibiotic |
| 114 | Improvement in prescribing habits and economic outcomes associated with the introduction of a standardized approach for surgical antimicrobial prophylaxis. | Hermsen et al., 2008 | The US | NS | CMA | No analysis on prophylactic antibiotic |
| 115 | Improving value in gynecologic surgery: We can all do better | Dowdy., 2013 | The US | Obstetric gynecology | CMA | No analysis on prophylactic antibiotic |
| 116 | Incidence and burden of surgical site infection for abdominal and vaginal hysterectomy using open and laparoscopic approaches | Waters et al., 2012 | The US | Obstetric gynecology | CMA | No analysis on prophylactic antibiotic |
| 117 | Infection control in the operating room. | Cosgrove., 2015 | The US | NS | CMA | No analysis on prophylactic antibiotic |
| 118 | Infection in vascular surgery: How to treat? | Henneberg., 2013 | Denmark | Cardiothoracic surgery | CMA | No analysis on prophylactic antibiotic |
| 119 | Initial treatment failure in patients with complicated skin and skin structure infections. | Berger et al., 2013 | The US | General surgery | CMA | No analysis on prophylactic antibiotic |
| 120 | Irrigation of abdomen with imipenem solution decreases surgical site infections in patients with perforated appendicitis: a randomized clinical trial. | Hesami et al., 2014 | Iran | General surgery | CMA | No analysis on prophylactic antibiotic |
| 121 | Is 6-week antibiotherapy effective in the treatment of early surgical site infections in instrumented spinal surgery? Preliminary results | Arvieu et al., 2016 | France | Orthopedic | CEA | No analysis on prophylactic antibiotic |
| 122 | Is Preoperative Nasal Povidone-Iodine as Efficient and Cost-Effective as Standard Methicillin-Resistant Staphylococcus aureus Screening Protocol in Total Joint Arthroplasty? | Torres et al., 2016 | The US | Orthopedic | CEA | No analysis on prophylactic antibiotic |
| 123 | Is preoperative screening for meticillin-susceptible Staphylococcus aureus in joint replacement surgery cost-effective to reduce surgical site infections? | Meda et al., 2016 | England | Orthopedic | CEA | No analysis on prophylactic antibiotic |
| 124 | Legacy health's 'big aims' initiative to improve patient safety reduced rates of infection and mortality among patients | Joyce et al., 2011 | The US | NS | CMA | No analysis on prophylactic antibiotic |
| 125 | Local application of gentamicin-containing collagen implant in the prophylaxis and treatment of surgical site infection following cardiac surgery. | Raja., 2012 | England | Cardiothoracic surgery | CMA | Review |
| 126 | Making a cost-effective contrast bone cement with or without antibiotics, using aqueous methylene blue for easy cement removal in revision and primary total joint arthroplasty. | Graves et al., 2007 | The US | Orthopedic | CEA | No analysis on SSI outcomes |
| 127 | Management of infections related to totally implantable venous-access ports: Challenges and perspectives | Lebeaux et al., 2014 | France | Oncosurgery | CMA | No analysis on prophylactic antibiotic |
| 128 | Managing healthcare resources: Estimated budget impact on the National Health service (England) of introduction of antimicrobial sutures | Edmiston et al., 2016 | The UK | NS | CEA | Review |
| 129 | Meta-analysis of the potential economic impact following introduction of absorbable antimicrobial sutures. | Leaper et al., 2017 | England | NS | CEA | Review |
| 130 | Natural coniferous resin salve used to treat complicated surgical wounds: Pilot clinical trial on healing and costs | Sipponen et al., 2012 | Finland | Orthopedic | CMA | No analysis on prophylactic antibiotic |
| 131 | Open-label clinical trial comparing the clinical and economic effectiveness of using a polyurethane film surgical dressing with gauze surgical dressings in the care of post-operative surgical wounds | Arroyo et al., 2015 | Spain | NS | CMA | No analysis on prophylactic antibiotic |
| 132 | Optimizing Value of Colon Surgery in Michigan. | Jaffe et al., 2017 | The US | General surgery | CMA | No analysis on prophylactic antibiotic |
| 133 | Outpatient parenteral antimicrobial therapy (OPAT) in the kingdom of bahrain: Efficacy, patient satisfaction and cost effectiveness | Al-Ansari et al., 2013 | Bahrain | NS | CMA | No analysis on SSI outcomes |
| 134 | Oxford craniotomy infections database: A cost analysis of craniotomy infection | O’keeffe et al., 2012 | The UK | Neurosurgery | CMA | No analysis on prophylactic antibiotic |
| 135 | Peri-operative antibiotic prophylaxis: adherence to guidelines and effects of educational intervention. | Ozgun et al., 2010 | England | NS | CMA | No analysis on prophylactic antibiotic |
| 136 | Perioperative antibiotic prophylaxis and cost in a Turkish University Hospital. | Yalcin et al., 2007 | Turkey | NS | CMA | No analysis on SSI outcomes |
| 137 | Perioperative closure-related complication rates and cost analysis of barbed suture for closure in TKA | Gilland et al., 2012 | The US | Orthopedic | CMA | No analysis on prophylactic antibiotic |
| 138 | Perioperative considerations of bilateral total knee replacement: A review | Gurunathan., 2013 | Australia | Orthopedic | CMA | Review |
| 139 | Perspective on the economic evaluation of deep brain stimulation | Mcintosh., 2011 | The UK | Neurosurgery | CMA | No analysis on prophylactic antibiotic |
| 140 | Plastic freezer bags: A cost-effective method to protect extraction sites in laparoscopic colorectal procedures? | Huynh et al., 2013 | Canada | General surgery | CMA | No analysis on prophylactic antibiotic |
| 141 | Postoperative fever. | Dionigi et al., 2006 | The US | NS | CMA | No analysis on prophylactic antibiotic |
| 142 | Predictors and costs of surgical site infections in patients with endometrial cancer | Bakkum-Gamez et al., 2013 | The US | Oncosurgery | CMA | No analysis on prophylactic antibiotic |
| 143 | Predictors of increased cost and length of stay in the treatment of postoperative spine surgical site infection | Blumberg et al., 2017 | The US | Orthopedic | CMA | No analysis on prophylactic antibiotic |
| 144 | Preoperative screening strategies for bacterialvaginosis prior to elective hysterectomy: A cost comparison study | Havrilesky et al., 2011 | The US | Obstetric gynecology | CMA | No analysis on prophylactic antibiotic |
| 145 | Preoperative use of mupirocin for the prevention of healthcare-associated Staphylococcus aureus infections: a cost-effectiveness analysis. | Young et al., 2006 | The US | NS | CEA | Review |
| 146 | Prescription of prophylactic antibiotics for neurosurgical procedures in teaching hospitals in Iran. | Askarian et al., 2007 | Iran | Neurosurgery | CMA | No analysis on SSI outcomes |
| 147 | Prevalence of hospital-acquired infections during successive surveillance surveys conducted at a University Hospital in The Netherlands | Hopmans et al., 2007 | The Netherlands | NS | CMA | No analysis on prophylactic antibiotic |
| 148 | Preventing infection in pediatric spinal fusion surgery: A novel perioperative and postoperative surgical site infection prevention bundle | Hennessey et al., 2012 | The US | Orthopedic | CMA | No analysis on prophylactic antibiotic |
| 149 | Prevention and control of surgical site infections: review of the Basel Cohort Study. | Junker et al., 2012 | Switzerland | General surgery | CMA | Review |
| 150 | Prevention, diagnosis, and treatment of gynecologic surgical site infections. | Lazenby et al., 2010 | The US | Obstetric gynecology | CMA | No analysis on prophylactic antibiotic |
| 151 | Process measures to improve perioperative prophylactic antibiotic compliance: quality and financial implications. | Sutherland et al., 2014 | The US | NS | CMA | No analysis on SSI outcomes |
| 152 | Prophylactic antibiotics for caesarean section: Which, when and why? | Ahmed et al., 2013 | The UK | Obstetric gynecology | CMA | No analysis on prophylactic antibiotic |
| 153 | Prosthetic joint infection: A microbiological review | Lalremruata., 2015 | India | Orthopedic | CMA | Review |
| 154 | Prosthetic joint infection: Recent developments in diagnosis and management | Cataldo et al., 2010 | Italy | Orthopedic | CMA | Review |
| 155 | Prosthetic joint infections - A need for health economy studies | Borgquist etal., 2014 | Sweden | Orthopedic | CEA | Review |
| 156 | Randomised clinical trial, observational study and assessment of cost-effectiveness of the treatment of varicose veins (REACTIV trial) | Michaels et al., 2006 | England | Cardiothoracic surgery | CUA | No analysis on prophylactic antibiotic |
| 157 | Recent advances in the prevention and management of complications associated with routine lumbar spine surgery. | Jenis et al., 2014 | The US | Orthopedic | CEA | Review |
| 158 | Reducing colon surgical site infections: A performance improvement project at two hospitals | Gabasan et al., 2011 | The US | General surgery | CMA | No analysis on prophylactic antibiotic |
| 159 | Reducing surgical site infections in hepatopancreatobiliary surgery. | Ceppa et al., 2013 | England | General surgery | CMA | No analysis on prophylactic antibiotic |
| 160 | Reduction in surgical antibiotic prophylaxis expenditure and the rate of surgical site infection by means of a protocol that controls the use of prophylaxis | Gomez et al., 2006 | Argentina | NS | CMA | No analysis on prophylactic antibiotic |
| 161 | Reduction of surgical site infection in spine surgery: An opportunity for quality improvement and cost reduction | McGirt et al., 2013 | The US | Neurosurgery | CMA | No analysis on prophylactic antibiotic |
| 162 | Reporting outcomesfrom antibiotic stewardship ward rounds: Does electronic referral hold the answer?category: Scientific free paper | Rivers et al., 2011 | The UK | NS | CMA | No analysis on prophylactic antibiotic |
| 163 | Rifamycin SV application to subcutanous tissue for prevention of post-cesarean surgical site infection | Kosus et al., 2010 | Nigeria | Obstetric gynecology | CMA | Included |
| 164 | Risk-adjusted increases in medical resource utilization associated with health care-associated infections in gastrectomy patients | Lee et al., 2010 | Japan | NS | CMA | No analysis on prophylactic antibiotic |
| 165 | Same-day discharge after appendectomy results in cost savings and improved efficiency | Farach et al., 2014 | The US | General surgery | CMA | No analysis on prophylactic antibiotic |
| 166 | Sequential antimicrobial treatment with linezolid for neurosurgical infections: efficacy, safety and cost study. | Martin-Gandul et al., 2016 | Austria | Neurosurgery | CMA | No analysis on prophylactic antibiotic |
| 167 | Simple, Safe, and Cost-Effective Technique for Resected Stomach Extraction in Laparoscopic Sleeve Gastrectomy. | Derici et al., 2016 | Egypt | General surgery | CMA | No analysis on prophylactic antibiotic |
| 168 | Single-dose metronidazole vs 5-day multi-drug antibiotic regimen in excision of pilonidal sinuses with primary closure: a prospective, randomized, double-blinded pilot study. | Chaudhuri et al., 2006 | Germany | General surgery | CMA | Included |
| 169 | Single-dose oral ciprofloxacin versus intravenous cefazolin prophylaxis in women undergoing midurethral sling surgery | Gomelsky et al., 2012 | The US | Urology | CMA | No analysis on SSI outcomes |
| 170 | Single-dose versus multiple-dose antibiotic prophylaxis for the surgical treatment of closed fractures. | Slobogean et al., 2010 | England | Orthopedic | CEA | No analysis on SSI outcomes |
| 171 | Single-incision laparoscopic appendectomy with a low-cost technique and surgical-glove port: "how to do it" with comparison of the outcomes and costs in a consecutive single-operator series of 45 cases | DiSaverio et al., 2016 | Italy | General surgery | CMA | No analysis on prophylactic antibiotic |
| 172 | Socioeconomic effects of surgical site infection after cardiac surgery in Japan | Kobayashi et al., 2014 | Japan | Cardiothoracic surgery | CMA | No analysis on prophylactic antibiotic |
| 173 | Surgical site infection in elderly patients with hip fractures, silver-coated versus regular dressings: a randomised prospective trial. | Kadar et al., 2015 | England | Orthopedic | CMA | No analysis on prophylactic antibiotic |
| 174 | Surgical site infection in spinal surgery: A comparative study between 2-octyl-cyanoacrylate and staples for wound closure | Ando et al., 2014 | Japan | Orthopedic | CMA | No analysis on prophylactic antibiotic |
| 175 | Surgical site infection prevention following total hip arthroplasty in Australia: a cost-effectiveness analysis. | Merollini et al., 2013 | Australia | Orthopedic | CEA | Included |
| 176 | Surgical site infection. Prevention and treatment | Santalla et al., 2007 | Spain | Obstetric gynecology | CMA | Review |
| 177 | Surgical site infections | Nel., 2014 | South Africa | NS | CMA | Review |
| 178 | Surgical site infections: does inadequate antibiotic therapy affect patient outcomes? | Eagye et al., 2009 | The US | NS | CMA | Lack of information on prophylactic antibiotics |
| 179 | Surgical treatment approaches and reimbursement costs of surgical site infections post hip arthroplasty in Australia: a retrospective analysis. | Merollini et al., 2013 | England | Orthopedic | CMA | No analysis on prophylactic antibiotic |
| 180 | Systematic review and cost analysis comparing use of chlorhexidine with use of iodine for preoperative skin antisepsis to prevent surgical site infection. | Lee et al., 2010 | The US | NS | CMA | Review |
| 181 | Systematic review of clinical and economic outcomes related to surgical site infections in Latin America | Charry et al., 2016 | Latin America | NS | CMA | Review |
| 182 | The "all-in-one" appendectomy: quick, scarless, and less costly. | Stylianos et al., 2011 | The US | General surgery | CMA | No analysis on prophylactic antibiotic |
| 183 | The clinical effectiveness and cost-effectiveness of enzyme replacement therapy for Gaucher's disease: A systematic review | Connock et al., 2006 | The UK | General surgery | CEA | Review |
| 184 | The cost effectiveness of vancomycin for preventing infections after shoulder arthroplasty: a break-even analysis. | Hatch et al., 2017 | The US | Orthopedic | CEA | No analysis on prophylactic antibiotic |
| 185 | The cost of infection in severe open tibial fractures treated with a free flap. | Olesen et al., 2017 | Germany | Orthopedic | CMA | No analysis on prophylactic antibiotic |
| 186 | The cost of vacuum-assisted closure therapy in treatment of deep sternal wound infection. | Mokhtari et al., 2008 | England | Cardiothoracic surgery | CMA | No analysis on prophylactic antibiotic |
| 187 | The cost-effectiveness of the DISINFECT Initiative (Decreasing the Incidence of Surgical INFECTions) in gynecologic oncology | Taylor et al., 2016 | The US | Obstetric gynecology | CEA | No analysis on prophylactic antibiotic |
| 188 | The economic burden of surgical site infection using therapeutic antibiotic utilization measure-comparison of two time periods | Patkar et al., 2010 | The US | General surgery | CMA | No analysis on prophylactic antibiotic |
| 189 | The economics and timing of preoperative antibiotics for orthopaedic procedures. | Norman et al., 2013 | England | Orthopedic | CMA | No analysis on prophylactic antibiotic |
| 190 | The effects of preoperative oral antibiotic use on the development of surgical site infection after elective colorectal resections: A retrospective cohort analysis in consecutively operated 90 patients. | Ozdemir et al., 2016 | The UK | General surgery | CEA | Included |
| 191 | The Epidemiology and Clinical Impact of Surgical Site Infections in the Older Adult | Bagdasarian et al., 2013 | The US | NS | CMA | Review |
| 192 | The epidemiology and cost of surgical site infections in Korea: a systematic review. | Lee et al, 2011 | Korea | NS | CMA | Review |
| 193 | The relationship between case volume, care quality, and outcomes of complex cancer surgery. | Auerbach et al., 2010 | The US | General surgery | CMA | No analysis on prophylactic antibiotic |
| 194 | The usage of 2-octyl cyanoacrylate post cesarean-section in canadian hospitals: A budget impact analysis | Goldstein., 2013 | Canada | Obstetric gynecology | CMA | No analysis on prophylactic antibiotic |
| 195 | The utilization of use of international evaluation indicator system of antimicrobials in inpatients | Qian et al., 2009 | China | NS | CMA | No analysis on prophylactic antibiotic |
| 196 | The Yin and Yang of pre-operative screening for meticillin resistant and sensitive Staphylococcus aureus (MRSA and MSSA): Does the extra effort and cost of suppression reduce surgical site infections? | Edmiston et al., 2013 | The UK | NS | CMA | Review |
| 197 | Treatment for peripheral arterial obstructive disease: An appraisal of the economic outcome of complications | Flu et al., 2008 | The Netherlands | Cardiothoracic surgery | CMA | No analysis on prophylactic antibiotic |
| 198 | Triclosan coated antibacterial suture: A budget impact analysis from Italian health service perspective | Velleca et al., 2014 | Italy | NS | CEA | No analysis on prophylactic antibiotic |
| 199 | Triclosan-coated sutures for surgical site infection prevention in mexican public health care institutions: Budget impact analysis | Zanela et al., 2016 | Mexico | NS | CEA | No analysis on prophylactic antibiotic |
| 200 | Triclosan-coated sutures for the reduction of sternal wound infections: economic considerations. | Fleck et al., 2007 | The Netherlands | Cardiothoracic surgery | CMA | No analysis on prophylactic antibiotic |
| 201 | Triclosan-coated sutures reduce the incidence of wound infections and the costs after colorectal surgery: a randomized controlled trial. | Nakamura et al., 2013 | The US | General surgery | CMA | No analysis on prophylactic antibiotic |
| 202 | UK DRAFFT: A randomised controlled trial of percutaneous fixation with kirschner wires versus volar locking-plate fixation in the treatment of adult patients with a dorsally displaced fracture of the distal radius | Costa et al., 2015 | The UK | Orthopedic | CMA | No analysis on prophylactic antibiotic |
| 203 | Usage of prophylactic antibiotics in uncomplicated gynecologic abdominal surgery in Siriraj hospital. | Boriboonhirunsarn et al., 2007 | Thailand | Obstetric gynecology | CMA | No analysis on SSI outcomes |
| 204 | Use of an observational, nationwide inpatient discharge database to document the economic benefits associated with innovative antimicrobial technology to reduce the risk of surgical site infection (SSI) | Edmiston et al., 2010 | The US | NS | CMA | No analysis on prophylactic antibiotic |
| 205 | Using a multi-faceted active change process and infection prevention to reduce post oP C-section infections | Harris et al., 2012 | The US | Obstetric gynecology | CMA | No analysis on SSI outcomes |
| 206 | Using PHMB antimicrobial to prevent wound infection | Moore et al., 2007 | The UK | NS | CMA | Review |
| 207 | Utilization pattern of antimicrobials among patients undergone midline laparotomy in four tertiary care teaching hospitals of Gujarat, India: Multi-centric retrospective study | Chadwa et al., 2013 | India | General surgery | CMA | No analysis on prophylactic antibiotic |
| 208 | Vascular catheter cultures for suspected catheter-related bloodstream infection in the ICU: A tradition whose time has passed? | Flynn et al., 2011 | The US | Urology | CMA | No analysis on prophylactic antibiotic |

NS, non-specific surgery; CMA, cost minimization analysis; CBA, cost benefit analysis; CEA, cost effectiveness analysis; CUA, cost utility analysis
