# Supplementary material for: Prevention of Surgical Site Infections: A Systematic Review of Cost Analyses in the Use of Prophylactic Antibiotics
Source: Front Pharmacol. 2018 Jul 18;9:776. doi: 10.3389/fphar.2018.00776 (PMC6060435; doi:10.3389/fphar.2018.00776)
Supplement: Supplementary file 4 [file Table_4.DOCX]

Supplementary Material

Prevention of Surgical Site Infections: A Systematic Review of Cost Analyses in the Use of Prophylactic Antibiotics

Abdul K.R. Purba^1,2,3*^, Didik Setiawan^4,5^, Erik Bathoorn^3^, Maarten J. Postma^1,2,4,6^, Jan-Willem Dik^3^, Alex W. Friedrich^3^

^1^Department of Health Sciences, University of Groningen, University Medical Center Groningen, Groningen, Netherlands

^2^Department of Pharmacology and Therapy, Universitas Airlangga, Faculty of Medicine, Surabaya, Indonesia

^3^University of Groningen, University Medical Center Groningen, Department of Medical Microbiology, Groningen, Netherlands

^4^Unit of PharmacoEpidemiology & Pharmacoeconomics (PE2), University of Groningen, Department of Pharmacy, Groningen, Netherlands.

^5^Department of Pharmacology and Clinical Pharmacy, Faculty of Pharmacy, Universitas Muhammadiyah Purwokerto, Purwokerto, Indonesia

^6^Department of Economics, Econometrics & Finance, University of Groningen, Faculty of Economics & Business, Groningen, Netherlands

*** Correspondence:**Abdul Khairul Rizki Purba

Email: [khairul_purba@fk.unair.ac.id](mailto:khairul_purba@fk.unair.ac.id)

# Supplementary Tables

**Table S4. PRISMA checklist**

| Section/topic | # | Checklist item | Reported on page # |
| --- | --- | --- | --- |
| TITLE | | |  |
| Title | 1 | Prevention of Surgical Site Infections: A Systematic Review of Cost Analysis in the Use of Prophylactic Antibiotics | Title |
| ABSTRACT | | |  |
| Structured summary | 2 | **Introduction**: The preoperative phase is an important period in which to prevent surgical site infections (SSIs). Prophylactic antibiotic use helps to reduce SSI rates, leading to reductions in hospitalization time and cost. In clinical practice, besides effectiveness and safety, the selection of prophylactic antibiotic agents should also consider the evidence with regard to costs and microbiological results. This review assessed the current research related to the use of antibiotics for SSI prophylaxis from an economic perspective and epidemiology of microbiological findings.  **Methods**: A literature search was carried out through PubMed and Embase databases from 1 January 2006 to 31 August 2017. The relevant studies which reported the use of prophylactic antibiotics, SSI rates and costs were included for analysis. The causing pathogens for SSIs were categorized by sites of the surgery. The quality of reporting on each included study was assessed with the “Consensus on Health Economic Criteria” (CHEC).  **Results**: We identified 208 eligible full-text studies reporting costs related to prophylactic antibiotics or SSIs. Three quarters (n=157) used cost-minimization analyses as the method of economic evaluation. Twenty of these 208 studies were included and were assessed subsequently, with the reporting quality scored on the CHEC list averaging 13.03 (8-18.5). Of these, 14 were trial-based studies, and the others were model-based studies. The SSI rates ranged from 0 to 71.1% with costs amounting to US$480-22,130. Twenty-four bacteria were identified as causative agents of SSIs. Gram negatives were the dominant causes of SSIs especially in general surgery, neurosurgery, cardiothoracic surgery and obstetric cesarean sections.  **Conclusions**: The fruitful information from some updated trial-based and model-based studies can be considered in the clinical implementation of the proper use of prophylactic antibiotics to prevent SSIs and antimicrobial resistance especially in terms of the cost and patterns of microbial causes of SSIs. Nevertheless, the findings of economics and microbiology from the included studies have reported diverse results.  Keywords: prophylaxis, surgical site infections, cost, bacteria” | Abstract |
| INTRODUCTION | | |  |
| Rationale | 3 | The preoperative phase is considered the most crucial period of a surgical procedure in which the goal is to reduce the bacterial load surrounding the incision area. Using antibiotics prior to surgical incision is considered to be effective in preventing SSIs, which are among the most common preventable post-surgery complications among healthcare-associated infections (HAIs) (Mangram et al., 1999; Umscheid et al., 2011). A parenteral prophylaxis agent spectrum with corresponding potential bacteria on particular sites of surgery has been recommended recently to reduce SSI rates efficiently (Berrios-Torres et al., 2017). In contrast, some preoperative procedures, such as hair removal and mechanical bowel preparation are considered today to be inefficient at reducing SSIs (Anderson et al., 2014; Leaper et al., 2008). In the US, SSIs were identified in approximately 1.9% of 849,659 surgical procedures in 43 states from 2006 to 2008 (Mu et al., 2011). The economic burden of SSIs should be taken into account in the use of prophylactic antibiotics. | Introduction |
| Objectives | 4 | The aim of this study is to present recent evidence and analyze the methodologies used in economic evaluations. In addition, the study comprehensively analyzes the quality of the included studies and local epidemiology of pathogen-causing SSIs. | Introduction |
| METHODS | | |  |
| Protocol and registration | 5 | This review was registered in PROSPERO with number CRD42017076589 | Materials and Methods |
| Eligibility criteria | 6 | We developed criteria to identify the eligible studies which contained economic analysis and followed the defined PICO-approach (Patient or Problem, Intervention, Control, and Outcomes). Concerning the patient (P), all types of surgical procedures were included. There was no restriction on age or gender. For both the intervention (I) and comparison (C), this review included studies concerning the utilization of antibiotic prophylaxis administered intravenously, orally, or locally to prevent SSI. Other terms of post-surgical infections such as wound infections and sternal wound infections (SWIs) were included. We excluded studies mainly evaluating comparisons of the use of antiseptic, pharmaceutical care interventions or guideline adherence issues. For the outcomes (O), we included studies evaluating both SSI rates and cost. | Materials and Methods (study selection) |
| Information sources | 7 | “We searched the updated relevant evidence from PubMed and EMBASE databases from 1 January 2006 to 31 August 2017. “ | Materials and Methods (search strategy) |
| Search | 8 | “The search strategy and the number of articles from PubMed and EMBASE are presented in S1 Table and S2 Table, respectively.” | Table S1 and Table S2 |
| Study selection | 9 | The search used search terms or phrases represented in Medical Subject Headings (MeSH) with the operator ‘tiab’ for PubMed. Subsequently, the terms or phrases used in PubMed were translated to the EMBASE database by using strings and the symbols ‘ab,ti’. To refine the result, we employed a search strategy using the Boolean operator ‘OR’ within sequences of terms with close or similar meanings and ‘AND’ for one or more sequences of terms which contained completely different meanings. Whole terms and phrases for either PubMed or Embase were identified by two persons (AKRP and KS) who dealt with the search strategy. | Materials and Methods (study selection) |
| Data collection process | 10 | Two authors (AKRP and DS) independently assessed all included papers. Any disagreements between those authors were discussed with a third author (JWD) until the discrepancies were resolved by consensus. | Materials and Methods (data extraction) |
| Data items | 11 | Fields of the extracted data included the authorship, year of publication, journal, country, type of surgery, wound categorization, gender, age, sample size, outcomes, prophylactic antibiotics, SSI rates, timing for the prophylactic strategy, follow-up and length of stay. To address the outcome from a microbiology perspective, we extracted the pathogens based on the sites of the surgery, antimicrobial susceptibility and their resistance rates. Furthermore, we grouped the types of SSIs based on the definitions and classifications of SSIs from the Centers for Disease Control and Prevention (CDC) (Horan et al., 1992). | Materials and Methods (data extraction) |
| Risk of bias in individual studies | 12 | We used the Consensus on Health Economic Criteria (CHEC) list to assess the quality of reporting of the health economic outcomes, including potential bias in individual studies (Evers et al., 2015). | Materials and Methods (quality assessments) |
| Summary measures | 13 | For the cost types, we took into account cost perspectives with components of (1) direct costs such as costs for prophylactic antibiotics, hospitalization, side-effects and antimicrobial resistance, and (2) indirect costs including costs of loss of productivity. We made costs comparable among individual studies using currency conversions to US$ and corrections for inflation rates. We calculated inflation rates based on the 2015 annual GDP growth index in the World DataBank for each respective country (The World Bank, 2015). If the individual article did not state the actual year for the cost analyses, we made the assumption that the year of the cost estimate was the same as the last year of data collection. | Materials and Methods (cost analysis and data synthesis) |
| Synthesis of results | 14 | - | - |
| Risk of bias across studies | 15 | - | - |
| Additional analyses | 16 | To address the outcome from a microbiology perspective, we extracted the pathogens based on the sites of the surgery, antimicrobial susceptibility and their resistance rates. Furthermore, we grouped the types of SSIs based on the definitions and classifications of SSIs from the Centers for Disease Control and Prevention (CDC) (Horan et al., 1992). | Materials and Methods (data extraction) |
| RESULTS | | |  |
| Study selection | 17 | This review initially identified a total of 644 and 1,417 articles from PubMed and Embase respectively. A comprehensive listing of the searches in both PubMed and Embase can be found in Table S1 and Table S2. After removing duplications, we screened 1,529 titles and abstracts. Subsequently, we excluded 1,321 articles for the reasons listed in the Materials and Methods section. Eventually, we assessed 208 eligible full-text studies of which we excluded 118 because of being reviews, having incomplete data related to costs and lack of presenting on the outcomes of prophylactic antibiotic uses and SSI incidence (Table S4). A total of 20 articles remained according to the inclusion criteria and were extracted systematically for further analyses (Alekwe et al., 2008; Chaudhuri et al., 2006; Courville et al., 2012; Dhadwal et al., 2007; Emohare et al., 2014; Gulluoglu et al., 2013; Joshi et al., 2016; Kosus et al., 2010; Matsui et al., 2014; Merollini et al., 2013; Patil et al., 2011; Theologis et al., 2014; Wilson et al., 2008). A flow chart of the search is shown in Figure 1. | Results and Figure 1. |
| Study characteristics | 18 | “General characteristics of 208 eligible and 20 included articles are presented in Table 1. Baseline overviews of country, types of surgery, gender, age, number of subject, types of prophylactic antibiotics, outcomes, design of the included studies are presented in Table 2.” | Table 1 and Table 2 |
| Risk of bias within studies | 19 | The range of CHEC scores in the included studies was from a low of 8 to a high of 18.5 (Dhadwal et al., 2007; Graves et al., 2016). The quality assessment scores of studies regarding general surgery ranged from 10 to 12 (Chaudhuri et al., 2006; Matsui et al., 2014; Ozdemir et al., 2016; Singh et al., 2014; Wilson et al., 2008). Among studies on orthopedic surgery and neurosurgery, the quality ranged between 12 and 18.5 (Ceballos et al., 2017; Courville et al., 2012; Elliott et al., 2010; Emohare et al., 2014; Graves et al., 2016; Lewis et al., 2016; Merollini et al., 2013; Theologis et al., 2014). Two cardiothoracic studies scored 8 and 11.5 points for CHEC items (Dhadwal et al., 2007; Joshi et al., 2016). Two obstetric and gynecological studies were scored at 10.5 and 11 (Alekwe et al., 2008; Kosus et al., 2010). Furthermore, two oncologic surgery studies obtained quality scores of 9.5 and 12.5 (El-Mahallawy et al., 2013; Gulluoglu et al., 2013; Patil et al., 2011). From the CHEC items, issues related to incremental analysis and sensitivity analysis were absent from most studies. The quality assessments of each article are reported in Table 6. | Results (quality assessments of included studies) and Table 6 |
| Results of individual studies | 20 | “Comparisons of included studies on reporting of cost index, methods of cost analysis, cost perspective, and adjusted cost in US$ at 2015-inflation rate are presented in Table 3.“ | Table 3 |
| Synthesis of results | 21 | - |  |
| Risk of bias across studies | 22 | With regards to new antibiotics, the pricing process has a significant influence on the calculation of the economic outcomes, and thus bias potentially comes particularly from trial-based economic studies that are sponsored by the pharmaceutical industry. The industry can affect the way in which results are reported (Bell et al., 2006; John-Baptiste and Bell, 2010). It is essential to adjust the costs for antibiotics especially for patented drugs that could decrease significantly in price when the patent period expires. Only 7(35%) included studies reported the costs of the antibiotics including the price of a single dose. Disclosure of either funding contributions or conflicts of interest in all the works and the findings of each study is a recommended strategy to identify bias (Palumbo et al., 2004). Half of the included studies explicitly included the statements of conflict of interest. In the economic evaluation, the outcome parameters are holistic including costs, clinical effectiveness and utility. Hence, a narrow or restricted perspective fosters omission of some essential costs and outcomes. Half of the included studies did not explicitly state the perspective, hence here may be cost measurement omission bias (Evers et al., 2005a). | Discussion |
| Additional analysis | 23 | From 7 included studies, this review generated a list of 24 bacteria that were reported as causing SSIs at the site of surgery on the cranium, thorax, abdomen, and thoracolumbar spine (Dhadwal et al., 2007; El-Mahallawy et al., 2013; Gulluoglu et al., 2013; Kosus et al., 2010; Lewis et al., 2016; Ozdemir et al., 2016; Theologis et al., 2014). The predominant species that have been reported to be found for SSIs were gram-negative bacteria. The common pathogen reported among studies was *Escherichia coli* isolates, accounting for 6.7-50% of incidence in general surgery, orthopedic, cardiothoracic surgery and cesarean section (Dhadwal et al., 2007; Kosus et al., 2010; Ozdemir et al., 2016; Theologis et al., 2014). More importantly, *Staphylococcus aureus* was the second most prevalent which was dominant among gram-positives causing SSIs (Dhadwal et al., 2007; El-Mahallawy et al., 2013; Gulluoglu et al., 2013; Ozdemir et al., 2016). Anaerobic bacteria were also reported, with an isolated case of *Bacillus fragilis* as a rare bacteria, accounting for approximately 13% of the SSI causes among cesarean section procedures (Kosus et al., 2010). We compiled the results of the pattern of bacterial causation of SSIs in Table 4. | Results (reports of the microbes causing SSI) |
| DISCUSSION | | |  |
| Summary of evidence | 24 | Obviously, CMA was simply used and implemented to address the costs due to the presence of SSIs such as in two studies in cesarean section and orthopedics which reported the median cost for SSIs at US$4,091 and US$108,782, respectively (Olsen et al., 2008; Thakore et al., 2015). The values were in line with the findings from the included studies which amounted to between US$482 and US$120,989. The high burden of post-surgical procedures when SSIs are concomitantly present with nosocomial pneumonia is also a complication post-surgery. The additional direct medical cost was considered to increase from EUR19,000 for SSIs to EUR35,000 for both post-surgical complications (Penel et al., 2008). Furthermore, in clinical outcome measurements, there is some evidence that systemic prophylactic antibiotics have a significant impact on minimizing the incidence of SSIs and medical costs in high-risk patients, especially in major surgical procedures including oncologic surgery (Jones et al., 2014), cardiothoracic (Lador et al., 2012), cesarean section (Smaill and Grivell, 2014) and orthopedic surgery (Brown et al., 2004). To achieve high efficacy, a current strategy is a prophylactic combination added locally to the standard prophylaxis, especially in deep surgical sites, for instance, using intra-wound vancomycin (Xiong et al., 2014)or gentamicin (Friberg et al., 2005). A meta-analysis showed that implantable gentamicin-collagen reduced either superficial or deep wound infection effectively, even though the mortality rate was not significantly different (Kowalewski et al., 2015). The use of a local or intra-wound antibiotic as an add-on treatment can be predicted as more effective since the site-target concentration of antibiotics with local treatment is higher than that without local antibiotics. In contrast, Eklund *et al*., stated that there was no statistically significant difference in SSI rates between an add-on local gentamicin group and the group without local prophylaxis (Eklund et al., 2005). | Discussion |
| Limitations | 25 | We are aware that this review may have limitations. For generalizability, the study is less representative for other important procedures such as urological, ophthalmological, or dental surgery. Using different definitions to determine SSI leads to underreporting of SSIs, even in community health services. The reporting of updated data related to microbiological results is fruitful, even though it may be more difficult to determine the definite cause of SSI at particular sites of incision from the results. Because of major differences in incidence of antibiotic resistance between the US and Europe, outcome studies need to be interpreted with caution. Finally, this review used the CHEC as a rigorous method to assess the quality of the articles and can be used as a baseline for guidelines for further economic evaluations (Evers et al., 2015). | Discussion |
| Conclusions | 26 | Overall, we describe novel findings from reviewing the economic evaluations of studies concerning prophylactic antibiotic uses for SSI prevention in general surgery, orthopedic surgery, neurosurgery, cardiothoracic surgery, obstetric and gynecological surgery, and oncologic surgery. Preoperative prophylactic antibiotics administered either locally or systemically are considered in some studies and for specific interventions at preventing SSIs. The quality in reporting of economic evaluation indicates that the included studies need to be improved for further research, especially with respect to issues related to antimicrobial susceptibility, pathogens causing SSIs, cost perspectives, incremental analysis and sensitivity analysis of the costs. Notably, the valuable information in terms of cost, updated causes of SSIs and local antimicrobial susceptibility from this review can be considered in the clinical implementation in the proper use of prophylactic antibiotics to reduce costs and to prevent SSIs and further antimicrobial resistance. | Conclusions |
| FUNDING | | |  |
| Funding | 27 | The work was supported by a Directorate General of Higher Education (DIKTI) scholarship, Ministry of Research, Technology and Higher Education of the Republic of Indonesia [No.224/D3.2/PG/2016]; and the Faculty of Medicine, Universitas Airlangga [No. 305/UN3.5/SDM/2016]; and Groningen University Institute for Drug Exploration, University Medical Center Groningen, The Netherlands. | Funding |

From: Moher D, Liberati A, Tetzlaff J, Altman DG, The PRISMA Group (2009). Preferred Reporting Items for Systematic Reviews and Meta-Analyses: The PRISMA Statement. PLoS Med 6(7): e1000097. doi:10.1371/journal.pmed1000097
